# pH-taxis drives aerobic bacteria in duodenum to migrate into the pancreas with tumors

**DOI:** 10.1038/s41598-022-05554-8

**Published:** 2022-02-02

**Authors:** Hiroaki Shirai, Cocoro Ito, Kosuke Tsukada

**Affiliations:** 1 Graduates School of Science and Technology, Keio University, 3-14-1 Hiyoshi Kohoku, Yokohama, Kanagawa 223-8522 Japan; 2 Department of Applied Physics and Physico-Informatics, Faculty of Science and Technology, Keio University, Yokohama, Japan

**Keywords:** Biomedical engineering, Pancreatic cancer

## Abstract

As oral or intestinal bacteria have been found in pancreatic cystic fluid and tumors, understanding bacterial migration from the duodenum into the pancreas via hepato-pancreatic duct is critical. Mathematical models of migration of aerobic bacteria from the duodenum to the pancreas with tumors were developed. Additionally, the bacterial distributions under the pH gradient and those under flow were measured in double-layer flow based microfluidic device and T-shaped cylinders. Migration of aerobic bacteria from the duodenum into pancreas is counteracted by bile and pancreatic juice flow but facilitated by pH-taxis from acidic duodenum fluid toward more favorable slightly alkaline pH in pancreatic juice. Additionally, the reduced flow velocity in cancer patients, due to compressed pancreatic duct by solid tumor, facilitates migration. Moreover, measured distribution of GFP *E. coli* under the pH gradient in a microfluidic device validated pH-tactic behaviors. Furthermore, *Pseudomonas fluorescens* in hydrochloride solution, but not in bicarbonate solution, migrated upstream against bicarbonate flow of > 20 μm/s, with an advancement at approximately 50 μm/s.

## Introduction

Prevention and treatments against pancreatic cancer with five-year survival rate 5–10% is urgent^1^. The pancreas, adjacent to and connected via hepatopancreatic duct to the duodenum, a part of the small intestine with abundant intestinal bacteria, attracts unique attentions to cancer researchers^2–14^, including a link between the oral microbiome and risk of pancreatic cancer^3^, oral bacteria found in pancreatic cystic fluid^4^, bacterial infection in chronic pancreatitis^5,6^. Moreover, human pancreatic ductal adenocarcinomas (PDACs) contain aerobic bacteria at higher levels than healthy pancreases^9–14^ (Table S1), which commonly favor neutral pH, such as *Pseudomonas putida*^15^, *Citrobacter freundii*^16^, *Klebsialla pneumoniae*^17^, and *Streptococcus*^18^. These bacteria in tumors contribute to cancer treatment^10–14^; for example, *Gammaproteobacteria* found in pancreatic cancer induced resistance to the widely used chemotherapeutic drug gemcitabine by converting it into inactive form with their enzymes^10^. Additionally, PDAC long-term survivors displayed diverse tumor microbes and immune activation^13^.

However, the mechanisms of bacterial migration into pancreatic tumors are poorly understood. A mechanistic understanding of bacterial migration from the duodenum into the pancreas in hepato-pancreatic duct is critical for both understanding the pancreatic disease and reducing infection. Previous findings demonstrated that bacterial DNA profiles in the pancreas of the same subjects were similar to those in the duodenum tissue^19^. In addition, orally administered *E. coli* was found in pancreatic tumors in mice^12^. However, bacterial invasion from the intestine into the pancreas is in general inhibited by the defense systems such as bile flow and the high-pressure zone at the sphincter of Oddi, a muscle situated at the junction of the duodenum and pancreatic duct that prevents the reflux from the duodenum^20^ (Fig. S1).

Mathematical modeling of bacterial penetration in the human gastrointestinal tract is missing in the literature. Bacterial penetration into meat and leafy vegetables with sessile drops were mathematically modeled previously^21–23^. Bacterial migration in colon mucus and to the epithelial layer was investigated^24^. Moreover, upstream swimming of *Escherichia coli* was analyzed in microfluidic devices^25–27^. Diao and coworkers developed a three-channel microfluidic device to analyze bacterial chemotaxis^28^.

Despite the aforementioned advances, a mechanistic understanding of the migration of aerobic bacteria from the duodenum into the pancreas with tumors has not been achieved. A solid tumor occurring at the pancreatic duct (PDAC) both reduces the function of pancreatic juice secretions and compresses the pancreatic duct and thus reduces pancreatic juice flow rates^29–31^. Moreover, pancreatic cancer is also frequently accompanied by biliary obstructions^32,33^. Thus a resistance of the flow to bacterial invasion is diminished. But bacterial random motility alone cannot win the fluid flow even at the velocity of cancer patients in literature^29–31^.

**Figure 1:**
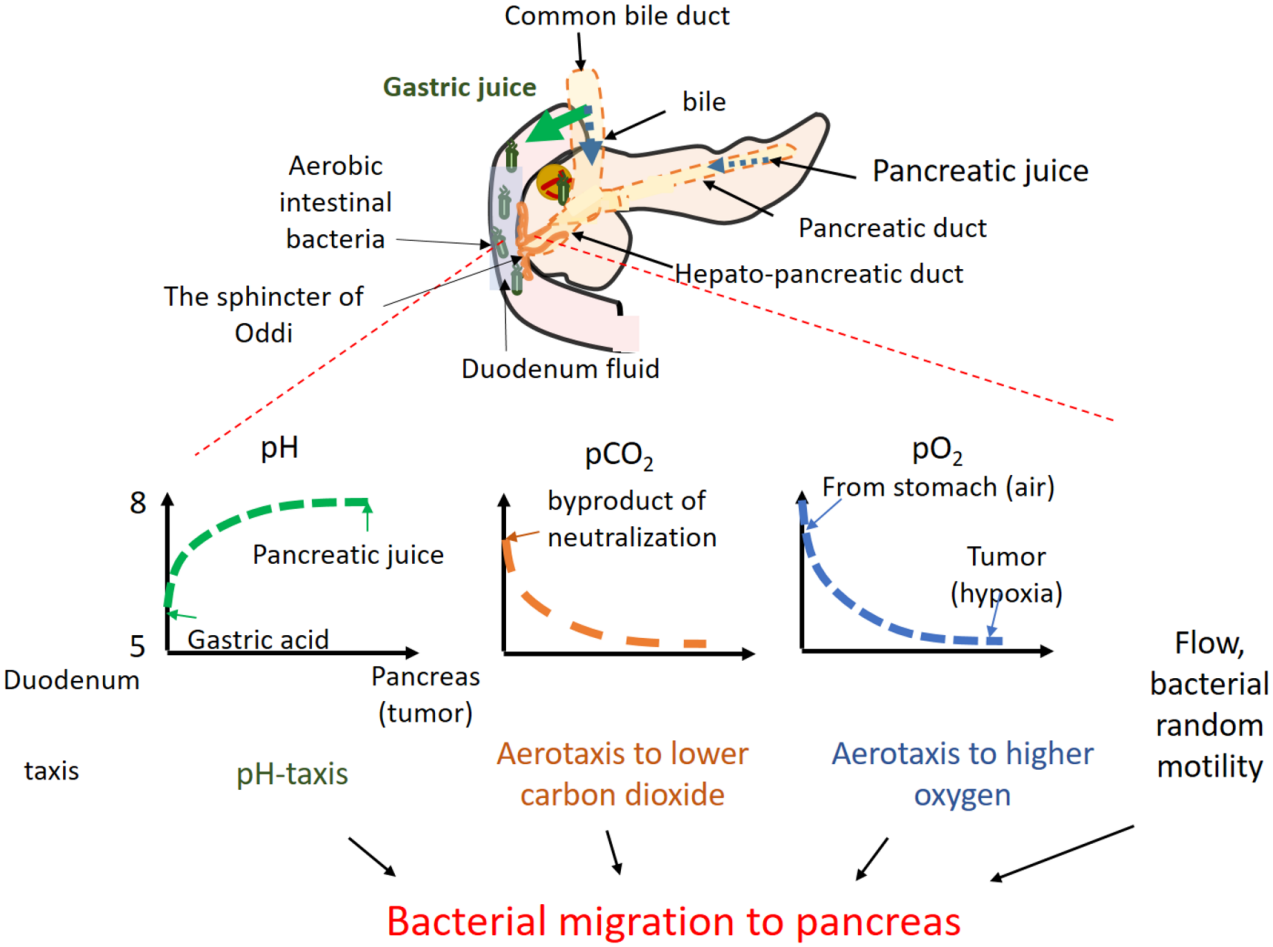
This work aims to understand how environmental factors in hepato-pancreatic duct such as pH, carbon dioxide and oxygen concentration, and fluid flow contribute to migration of aerobic bacteria from the duodenum to the pancreas with tumors.

Motile bacterial cells with flagellar such as *Pseudomonas putidai and Citrobacter freundii* show random ‘run’ and ‘tumble’ motility by rotating their flagellar counter-clockwise (run) and clockwise (tumble) without any attractant^34^. However, with attractant or repellent that they sense through the chemo-receptors, bacteria ‘tumble’ less and thus ‘run’ more in swimming toward more favorable environment, showing called ‘tactic’ behaviors^35^. Thus the migration of aerobic bacteria from the duodenum to pancreas with tumors is influenced by the environmental factors at upper gastrointestinal tract such as pH, carbon dioxide and oxygen concentration via their sensing mechanisms of pH- and aerotaxis^36,37^ (Fig. 1).

The hypotheses of this work are twofold: (1) migration of aerobic bacteria from the duodenum into the pancreas is explained by a mathematical model that includes bacterial random motility, pH taxis from acidic environment to neural one, aerotaxis to higher oxygen and lower carbon dioxide, and the pancreatic juice and bile flow, and (2) bacterial migration from the duodenum to the pancreas in the hepatopancreatic duct is experimentally modeled in a T-shaped cylinder, simply mimicking the pancreatic duct. The outlines of this work is as follows. First the simulated migrations of aerobic bacteria from the duodenum to the pancreas with tumors are provided. Second, the pH-tactic behaviors of *GFP E. coli* were demonstrated in a pH-gradient reproducible microfluidic device. Finally, pH-tactic upstream migrations of *P. fluorescens* from the acidic solution against neutral flow are provided and the models are validated. This work aims to understand how each factor and its combination with others contribute to the migration of aerobic bacteria from the duodenum to the pancreas with tumors.

## Results

### Mathematical modeling demonstrate that the pH-taxis under the pH-gradient between acidic duodenal fluid and slightly alkaline pancreatic juice drives aerobic bacteria in duodenum to migrate into pancreas

**Figure 2:**
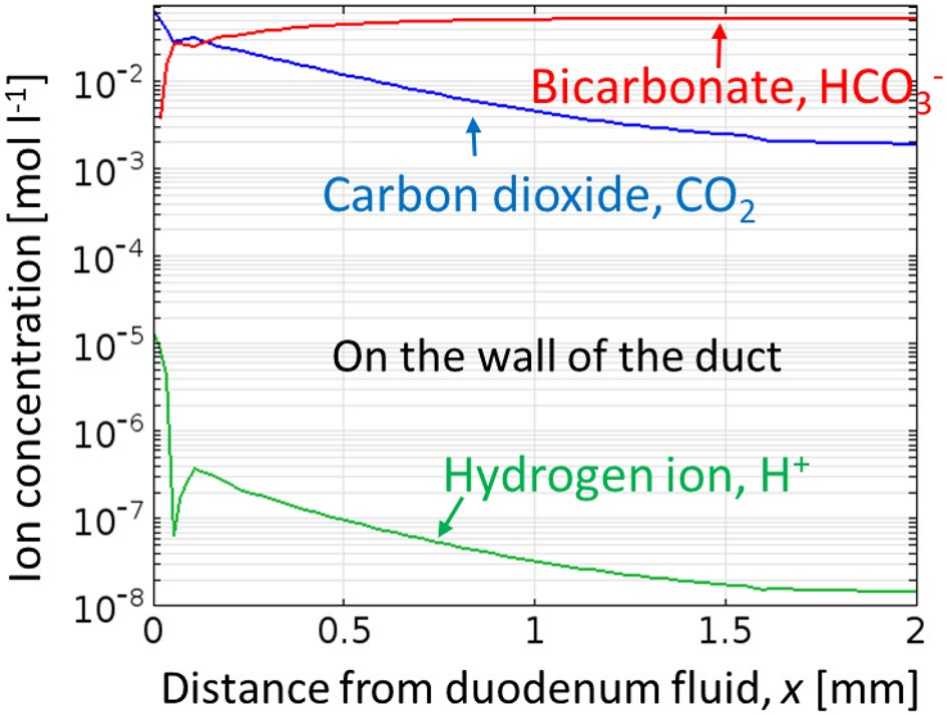
Simulated concentrations of carbon dioxide (blue), hydrogen ion (green), and bicarbonate (red) on the wall of hepato-pancreatic duct. Simulated pH increased greatly between the duodenal fluid and the pancreatic duct (green) since hydrogen ions (green) were neutralized by bicarbonate (red) with carbon dioxide as a byproduct (blue). Simulated ion concentration distribution in the hepatopancreatic duct of *healthy* individuals. Ion concentrations at a ductal radius of 2.6 mm $pH = - \log_{10} ([H^{{_{ + } }} ])$.

The simulated pH in the hepatopancreatic duct increased greatly between the duodenum with lower pH and the pancreas at neutral or slightly alkaline pH (Fig. 2 green, Eqs. 14 and 15). This is because the diffusion of gastric acid in duodenum (at pH 5–6)^38^, originally from stomach, into the duct is neutralized by bicarbonate in pancreatic juice. In addition, carbon dioxide is generated here as a byproduct of neutralization (Eqs. 14 and 15, Fig. 2 blue). The simulated pH in the pancreas at 7.6 (Fig. 2 green) agrees reasonably well with the literature that pancreatic juice has a pH of 8.0–8.3 and liver bile has pH at 7.8^39^.

**Figure 3:**
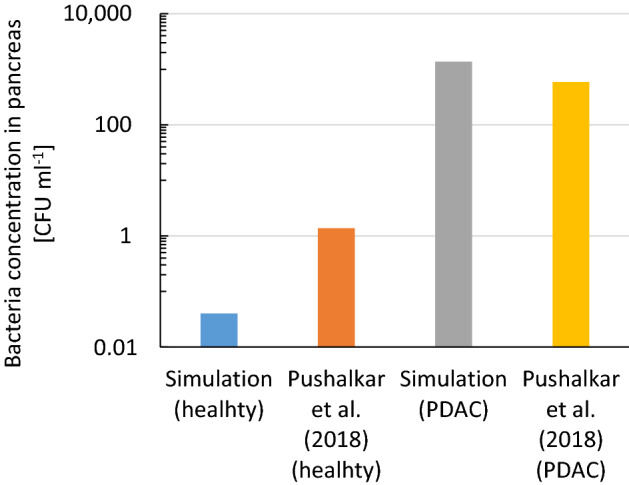
The simulated bacterial concentration in the healthy pancreas (blue) is lower than that in the literature (orange), but simulated bacterial amount in pancreas at 3.2 CFU agrees reasonably we the literature that 15% of healthy pancreas contained detectable bacteria^10^. Simulated one in the pancreas with tumors (gray) agrees reasonably well with the literature^12^ (yellow). The literature value was calculated using the DNA weight of *E. coli* at 17 fg/cell. PDAC: pancreatic ductal adenocarcinoma.

The migration of aerobic bacteria in the hepatopancreatic duct was then simulated (Figs. 3, S2). Factors that influence bacterial transport are summarized in Table 1. Although the simulated bacterial concentration in the healthy pancreas (Fig. 3 blue) was lower than that in the literature (Fig. 3 orange)^12^, the bacterial amount estimated using the typical weight of the pancreas at 80 g at 3.2 CFU seems consistent with the literature that 15% of healthy pancreas contained detectable bacteria^10^. Bacterial migration in hepatopancreatic duct due to bacterial random motility alone is limited (Fig. S2b black dotted). But this migration is greatly facilitated by bacterial pH-taxis from acidic duodenum fluid into pancreatic duct containing pancreatic juice at slightly alkaline pH (Figs. 2 green, S2a, b blue and green). Note aerotaxis of aerobic bacteria away higher carbon dioxide concentration at the duodenum affects migration little since the pH-tactic velocity is approximately tenfold larger than aerotactic velocity (Fig. S2b blue and red).

**Table 1:** List of the factors that influence transports included in this work.

Motility	Run-and-tumble random motion using flagellar, increases migration
Chemotaxis	Migration toward chemo-attractant or away from repellent
Aerotaxis	Energy taxis, toward higher oxygen (decreases migration) and away from higher carbon dioxide (increases migration)
pH-taxis	Migration from acid or alkaline pH toward neutral one, increases migration

### Bacterial migration from the duodenum to pancreas with tumors is made easier by the obstruction of pancreatic and bile ducts

Oxygen transport in pancreas with tumors was simulated to investigate the role of aerotaxis toward higher oxygen at duodenum (Eqns. S7–S9). Oxygen dissolved in duodenal fluid, from stomach fluid in equilibrium to air, diffuses into both the hepatopancreatic duct and duodenal wall, and toward the pancreas with the tumor, with the lower oxygen concentration due to tumor hypoxia (Fig. S4). Note that diffusion in the duct is inhibited by flow, while that in the duodenum wall is not inhibited but by the physical barrier of the wall (Fig. S4). Additionally, oxygen in the duodenal wall diffuses into the duct through the wall of the duct, leading to higher oxygen concentration near ductal wall (Eq. S9) (Fig. S4).

The simulated bacterial concentration in the pancreas with tumors (Fig. 3 gray) was over 100 times higher than that in the healthy pancreas (Fig. 3 blue) and agreed reasonably well with the literature (Fig. 3 yellow). This higher concentration in pancreas with tumors than healthy pancreas is also consistent with previous literature that 83% of pancreatic tumors contained detectable bacteria, while bacteria were detected in just 15% of healthy pancreas^10^. Easier bacterial migration to pancreas with tumors than to healthy pancreas (Fig. S2, 4 and S4) is due to the reduced pancreatic juice and bile flow rates, caused by obstructions of pancreatic and bile ducts^29–31^. Additionally, the bacterial concentration in the pancreas with tumors was ellipse-shaped with a lower concentration along the pancreatic duct and a higher concentration along the duodenum wall (or bile duct), due to reflux back to the duct with flow (Fig. S4).

**Figure 4:**
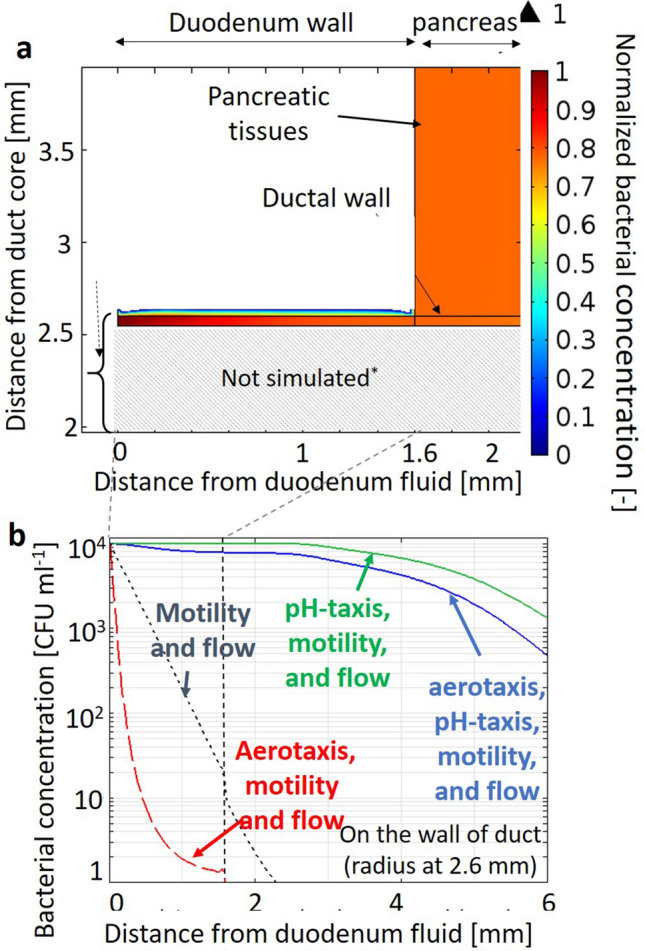
Simulated bacterial concentrations in hepato-pancreatic duct and surrounding pancreatic tissues in cancer patients (**a**) and those on the wall of the duct (**b**). The major driving force of bacterial migration is pH-taxis from acidic to neutral pH. This migration is made easier by reduced pancreatic juice and bile flow rate due to obstructions of the pancreatic and bile duct by solid tumors.

Aerotaxis of aerobic bacteria toward higher oxygen at duodenum (Fig. S5) had little effect on migration (figure S5). This is because the aerotaxis of aerobic bacteria to higher oxygen at the duodenum is outweighed by pH-taxis to neutral pH (Fig. S5). Thus, aerobic bacteria, showing aerotaxis to higher oxygen, even migrated into the pancreas with the tumor (Fig. S5blue).

### Parametric sensitivity analysis

**Figure 5:**
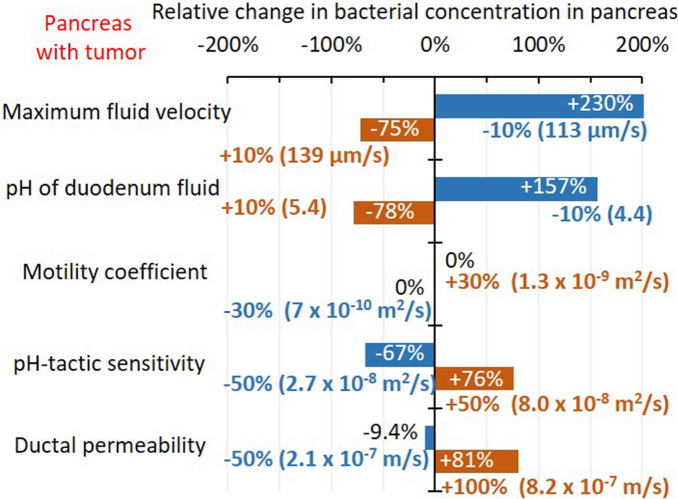
Parametric sensitivity analysis for bacterial migration from the duodenum into the pancreas with tumors. The changes in simulated bacterial concentration in pancreas are shown with each parameter changed.

Bacterial migration to pancreas with tumors was simulated when each parameter of bacterial transport such as maximum fluid velocity, *v*_*max*_ [μm/s], duodenal pH [−], bacterial random motility coefficient, $\mu_{0}$ [m^2^ s^–1^], pH-tactic sensitivity, $\chi_{0}^{pH}$, and ductal permeability, *P*_*b*_ [m s^–1^] are changed to a certain extent to investigate how each parameter influences bacterial concentration in pancreas more. Note the simulated bacterial concentration were very sensitive to maximum fluid velocity and pH of duodenum, thus these parameters were change by 10%, while other ones were changed by 30–100%. Increased maximum fluid velocity greatly reduced simulated bacterial concentration in the pancreas (Fig. 5). Increased pH of duodenal fluid also reduced bacterial concentration in the pancreas greatly by decreasing the pH gradient between the duodenum fluid and pancreatic juice (Fig. 5). The random motility coefficient has no effect on penetration, although the pH-tactic sensitivity coefficient greatly affects migration (Fig. 5). Increased permeability of the hepatopancreatic duct also increased migration to the pancreas by increasing efflux from the duct to pancreatic tissues (Fig. 5).

### Measured migrations of GFP *E. coli* under the pH gradient in a microfluidic device validates pH-taxis

**Figure 6:**
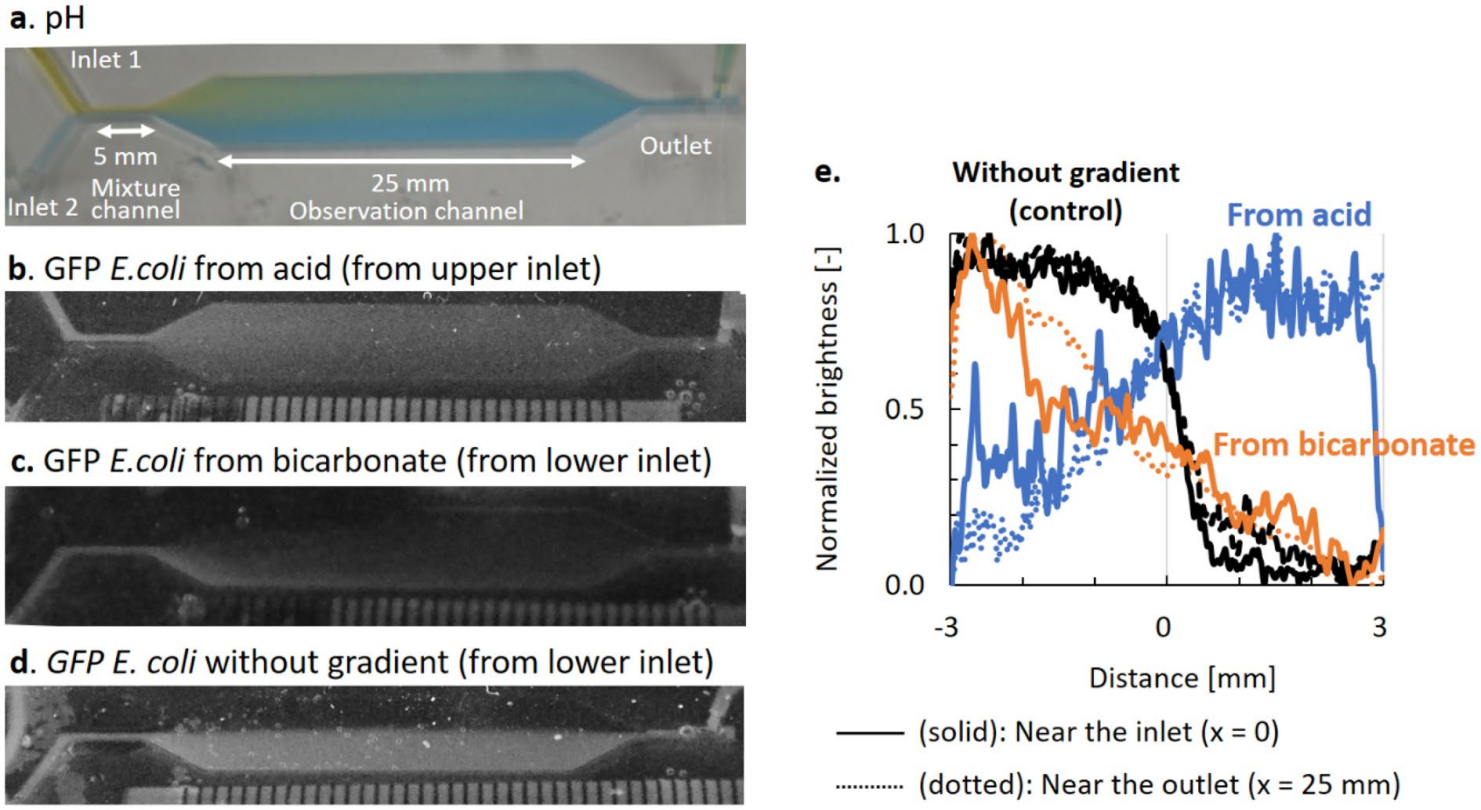
A steady pH gradient is generated in a microfluidic device, with pH at 5–6 at the top and 7–8 at the bottom (**a**). GFP *E. coli* were attracted away from the upper channel with a lower pH toward the lower part with neutral pH (**b**, **c, e** blue and orange), though bacteria migrated vertically little without gradient (**d**). **a**: pH was visualized in bromothymol blue. **b**, **c**: Bacteria were included in either the upper inlet (**b**) or the lower inlet (**c**). **d**: GFP *E.coli* migrated little without gradient. **e**: Distribution of GFP *E. c*oli in the proximal (dotted) and distal (solid) channels*.* Photos in **b**–**d** were taken in black-and-white mode under black light at 350 nm.

The pH-tactic migrations of bacteria were measured using a pH-gradient generating double-laminar flow-based polydimethylsiloxane (PDMS) microfluidic device (Fig. 6a). When bicarbonate and hydrochloride solution were poured in the top and bottom inlet, respectively, a steady pH gradient is generated in the channel with pH at 5–5.5 on the top and at 8–9 at the bottom (Figs. 6a, S6). When GFP *E. coli* were included only in the upper channel without gradient as a control, GFP *E. coli* migrated little without a gradient with a diffusion-like distribution (Fig. 6d, e black). The calculated root-mean-square displacement: $x = \sqrt {2\mu t}$, with random motility coefficient, $\mu$ [m^2^ s^–1^] (Eq. 6) due to motility alone within the 25 mm-length channel is 300 μm. This migration length seems to be much smaller than the width of the channel of 6 mm, and thus consistent to the little differences in measured distribution of bacteria at the proximal (Fig. 6e black, dotted) and far areas (Fig. 6e black, solid). However, when GFP *E. coli* are poured at the upper inlet with hydrochloride solution under the pH-gradient, bacterial distribution was more heterogeneous (Fig. 6b, d, e blue), indicating migration from upper area with lower pH toward lower neutral or slightly alkaline area, due to pH-taxis. On the other hand, when including bacteria at the lower inlet with bicarbonate solution, bacterial distribution is limited at the lower channel, where pH is neutral or alkaline (Fig. 6c, e orange).

When *Pseudomonas fluorecens* was used as bacteria in the same conditions to compare with *E. coli*, bacterial distribution seemed to be biased at central areas, where pH is closer to neutral (Figs. 6a, S6, and S7). This may possibly be due to the difference in optimal pH, as *E. coli* have optimal pH at 8^36^, while that of *P. fluorescens* was at 7.5. Note that carbon dioxide was generated at the top channel (0.7 mmol l^–1^) due to neutralization (Fig. S8), where *E. coli* was attracted toward higher carbon dioxide^40^. Thus, a higher bacterial concentration in the lower channel, where the carbon dioxide concentration is lower (Figs. 6b, c, e orange and green, S8), still assures pH taxis.

### Measured upstream migration of *P. fluorescens* in a T-shaped cylinder validates the models

Upstream migrations of *Pseudomonas fluorecens* in a four-millimeter T-shaped cylinder against the bicarbonate flow at 20 μl/min were measured to validate the models (movies S1 and S2). Bicarbonate solution was put in equilibrium to 5% carbon dioxide in advance to make the pH at the same level as in pancreas (Fig. S9). *Pseudomonas fluorescens* was chosen here, as *Pseudomonas* was one of the most commonly found strains in pancreatic cancer^10^ (Table S1) and visible with their intrinsic fluorescence under UV light. A lower flow rate of 20 μl/min was chosen to easily observe bacterial migration under flow conditions.

**Figure 7:**
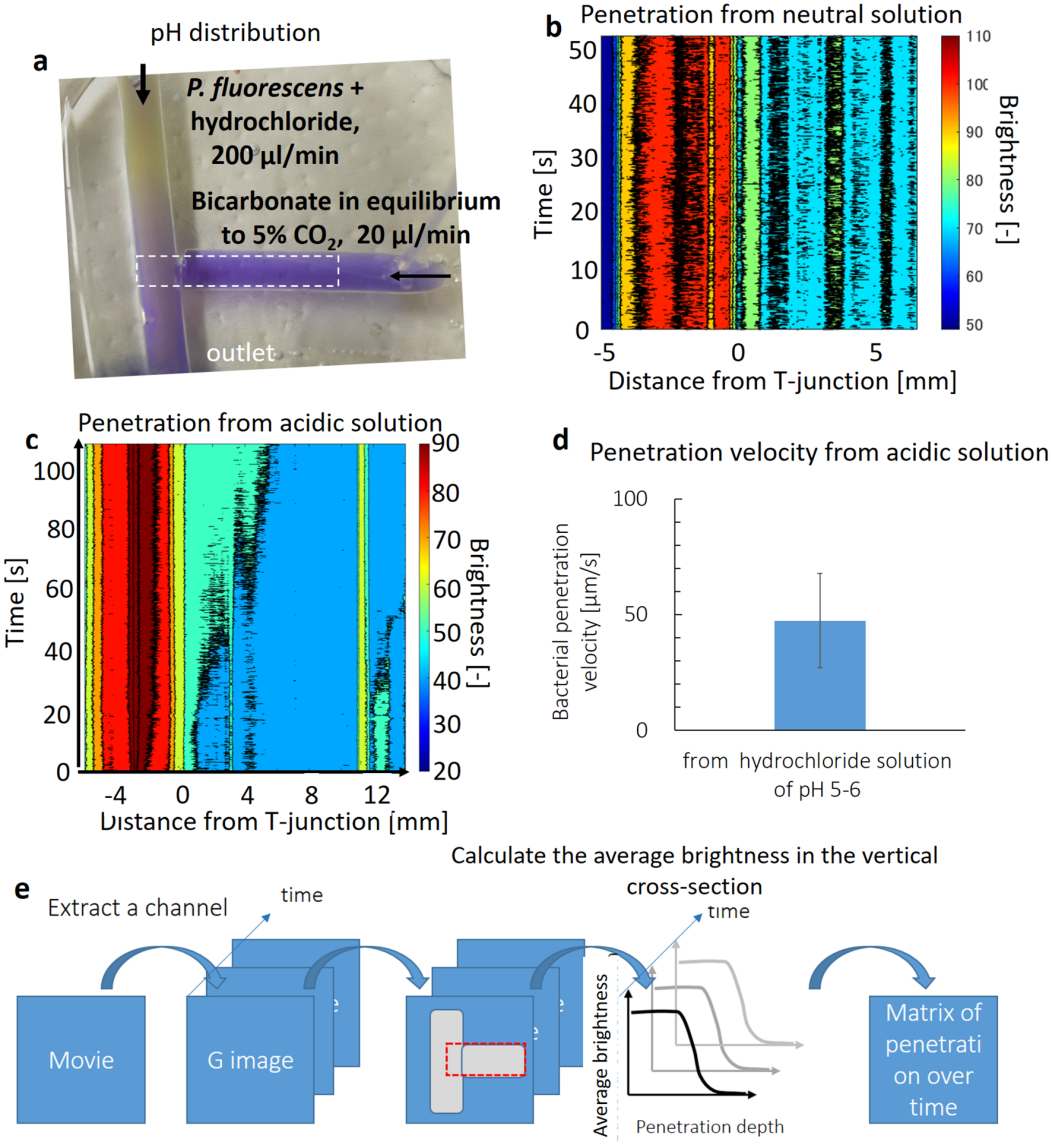
Migration of *P. fluorescens* from hydrochloride solution against the bicarbonate flow of μl/min was measured in a simply fabricated PDMS four-millimeter diameter T-shaped cylinder. **a**: Distribution of pH in the T-shaped cylinder is visualized in bromocresol purple. The pH increased from 5–6 in dark yellow at the top to purple (neutral) at the right and the bottom of the cylinder. **b**: An example of bacterial distribution over time from bicarbonate solution as a control. **c:** *P. fluorescens* migrated upstream against flow under the pH gradient at the T-junction (**a**) with a penetration rate of approximately 50 μm/s. **d**: Average bacterial penetration velocity in three different experiments. **e:** A procedure for calculating a matrix of bacterial penetration depth over time (**b**, **c**). Bacterial distribution was quantified from the brightness of the image of each frame in the movies, under UV light in dark condition, which corresponds to the intrinsic fluorescence of *P. fluorescens*.

When *P. fluorescens* were included in bicarbonate solution at the left cylinder as a control, bacterial distribution in the right cylinder did not change over 50 s, suggesting no upstream migration without pH-gradient (Figs. 7b, S10b, and movie S1). This agrees with the simulated migrations that the bacterial concentration due to motility alone dropped to 1/100 at one millimeter after five minutes (Fig. S11b). Note the vertical axis is logarithmic scale (Fig. S11b). Then *P. fluorescens* were included in hydrochloride solution of pH 5–6 to analyze the roles of pH-gradient in the migrations. A pH-gradient was observed at the T-junction of the cylinder, with the pH at 5–6 (dark yellow) at the top and neutral (purple) at the right and bottom (Fig. 7a). In the pH-conditions, *P. fluorescens* migrated upstream against bicarbonate flow (Figs. 7c and S10a, movie S2), with the penetration velocity at approximately 50 μm/s (Figs. 7c, d and S10a). pH taxis of *P. fluorescens* drove them from acidic solution into neutral pH areas (Fig. 7a), which wan fluid flow in the cylinder, near the wall with lower fluid velocity (movie S2). This migration seems to be heterogeneous (movie S2), due to a parabolic fluid flow velocity distribution in the cylinder (Eq. 18, Hagen-Poiseuille law). Additionally, migration immediately close to the T-junction is swift, due to a greater pH gradient (Fig. 7a), while advancement in the areas far from the junction is slower (Fig. 7c, S10a, movie S2). This also agrees reasonably well with the simulation results, validating the models (Fig. S11a, movies S3 and S4).

## Discussion

### Factors contributing to faster pH-tactic velocity

The measured pH-tactic velocity in a T-shaped cylinder at approximately 50 μm/s (Fig. 7d) is much faster than the typical chemotactic velocity of 10 μm/s, due to the following reasons. First, the gradient under flow is made greater since the flow inhibits diffusion (Fig. 2 green). Second, pH is logarithm of hydrogen ion concentration. Thus an increase in pH leads to an exponential decrease in concentration of hydrogen ion. Therefore, the pH-tactic velocity, influenced by the logarithm of concentration gradient (Keller-Segel model), should be faster.

The pH-tactic velocity, *v*_*pH*_*(h)* can be quantified with the fluid velocity, *v*_*f*_*(r)* [μm/s] (eqns.18 and 19) and measured penetration velocity in the cylinder, *v* [μm/s], which can be approximated from the slope of the Fig. 7c, $v = v_{pH} (h) - v_{f} (r)$. Note aerotactic velocity and motility are considered to be negligible to pH-taxis, as described above. The penetration into pancreas occurs when *v* > 0, i.e., $v_{pH} (h) > v_{f} (r)$ . As the fluid velocities in the duct are parabollic (Eqs. 18 and 19), the fluid velocity should be equal to the pH-tactic velocity at a certain radius at *r*_*b*_, satisfying $v_{pH} (h) = v_{f} (r_{b} )$, Thus the followings are obtained :

1 $$\begin{aligned} & v_{pH} (h) > v_{f} (r) \, \;{\text{ (r}}_{{\text{h}}} {\text{ > r > r}}_{{\text{b}}} {)} \\ & v_{pH} (h) < v_{f} (r) \, \;{\text{(r < r}}_{{\text{b}}} {)} \\ \end{aligned}$$

Note the radius, *r*_*b*,_ is calculated from Keller-Segel model^41^ and flow velocity (Eqs. 18 and 19) as:

2 $$\overbrace {{ - \chi \frac{1}{h}\frac{dh}{{dx}}}}^{{\text{pH - taxis}}} = \overbrace {{\frac{{2Q_{h} }}{{\pi r_{h}^{2} }}\left\{ {1 - \left( {\frac{{r_{b} }}{{r_{h} }}} \right)^{2} } \right\}}}^{{{\text{flow}}}}$$

This equation can be simplified as:

3 $$r_{b} = r_{h} \sqrt {1 - \frac{{v_{pH} }}{{v_{\max } }}}$$

This can be re-written with pH-tactic sensitivity coefficient, pH-gradient, cross-section areas of the duct, and fluid flow rate as input parameters:

4 $$\frac{{r_{b} }}{{r_{h} }} = \sqrt {1 - \frac{{\overbrace {2.3}^{\substack{ {\text{conversion of}} \\ {\text{ logaithm}} } }}}{2} \cdot \overbrace {{\left( { - \underbrace {\chi }_{\begin{subarray}{l} {\text{chemoatctic }} \\ {\text{sensitivity}} \\ {\text{coefficient}} \end{subarray} }\underbrace {{\frac{d(\log (h))}{{dx}}}}_{{\text{pH - gradient}}}} \right)}}^{{\text{pH - tactic velocity}}} \cdot \frac{{\overbrace {{\pi r_{h}^{2} }}^{\substack{ {\text{cross - section}} \\ {\text{area}} } }}}{{\underbrace {Q}_{{\text{flow rate}}}}}}$$

Note the pH-gradient is dependent on the distance from the duodenum (Fig. 2 green), thus the ratio, *r*_*b*_*/r*_*h,*_ increases with the distance from duodenum. Additionally, the ductal radius, *r*_*h*_ is smaller at the orifice of major papilla and larger in the duct close to pancreas^42^. Smaller radius of the duct, *r*_*h,*_ leads to increased ratio, *r*_*b*_*/r*_*h,.*_ Note as most cases in healthy individuals, possible bacterial entry into pancreas is flushed away by pancreatic juice flow (Fig. S2), decrease in fluid flow rate., *Q,* or lower duodenal pH, −log(*h*)) seems needed for the bacterial entry in pancreatic cystic fluid^3,4^ or tumors^10,12^.

### Origins of bacteria in pancreatic tumor

Previously proposed origin includes the duodenum (small intestine) via the pancreatic duct and large intestine through the portal vein^11^. Since pancreatic cancer contains immotile bacteria (table S1) without motility or pH-taxis that do not migrate into the pancreas even under the reduced flow (Fig. 4 black), the latter route, i.e., from large intestine, is not neglected. Moreover, impaired intestinal barrier in patients with obstructive jaundice, which is accompanied by pancreatic cancer^32^ promote bacterial translocation via the bloodstream^43^. However, bacterial colonization in the pancreas was not detected in a mouse model with defective intestinal permeability with increased permeability by *Campylobacter* infection^7^. The former route is justified by motile and highly aerobic bacterial strains found in pancreatic tumors such as *Pseudomonas putida* and *Citrobacter*^10^ (Table S1) as oxygen concentration in duodenum is relatively higher and bacteria in colon are typically obligate anaerobes. Also previous findings that orally administered *E. coli* were found in pancreatic tumors also agree with this route^12^.

### Pathway for migration of aerobic bacteria to pancreatic tumors

The pathway for migration of aerobic bacteria from the duodenum into the pancreatic tumor is divided into the following four: (i) at the T-junction of duodenum and pancreatic duct, i.e., high pressure zone of the sphincter of Oddi, driven by pH-taxis under a sharp pH-gradient (Figs. 2 green and 4), (ii) in the hepato-pancreatic duct, driven by pH-taxis under a milder gradient (Figs. 2, 4, S2, S3), (iii) through the ductal wall out to pancreatic tissues (Figs. 4 and S2), (iv) in pancreatic interstitium and tumor one (Fig. 4, S3). The first step is made easier in cancer patients with obstructions of the bile and pancreatic duct (Figs. 2, 4, and S3). The second step is in the duct in the duct with flow in duodenum wall. The third step is probably driven by the concentration difference between the duct and the interstitium (Eq. 10). The last step is migration in interstitium, where bacterial motility is not inhibited by the flow but by the geometric barrier of the interstitium as a porous medium. Bacterial motility in tumors with densely packed interstitium is more reduced^44^. Note that bacteria in healthy tissues are probably at the end eliminated by the immune system, while those in tumors are not due to the suppressed immune system^45^.

This mechanistic understanding is relevant to all possible transport phenomena between duodenum and pancreas, such as a link between oral microbiome and risk of pancreatic cancer^3,4,46^, roles of bacteria in carcinogenesis^7,8^, bacterial infection on common bile duct^33^ and in pancreas with chronic pancreatitis^6^, and bacteria in pancreatic tumor affecting chemo- or immunotherapy^10,12^. For example, possible entry of oral bacteria in the duodenum into the healthy pancreas (Fig. S2) might possibly be associated with cancer risk^3,4,46^. Additionally, infection in pancreas with chronic pancreatitis may possibly be linked to not just reduced flow rate^30^ but also acidified duodenum^47^, caused by insufficiency of bicarbonate secretion^6^ as lower duodenal pH increases the pH gradient between the duodenum and the pancreas (Fig. 5). In terms of cancer treatment, reducing bacterial migration into the pancreas with tumors may help antibiotic strategies improve the efficacy of gemcitabine^10,48,49^. Moreover, clinical translation of the fecal microbial transplant (FMT) strategy to directly or indirectly influence the tumor microbiome^13,50^ might benefit.

## Conclusion

**Figure 8:**
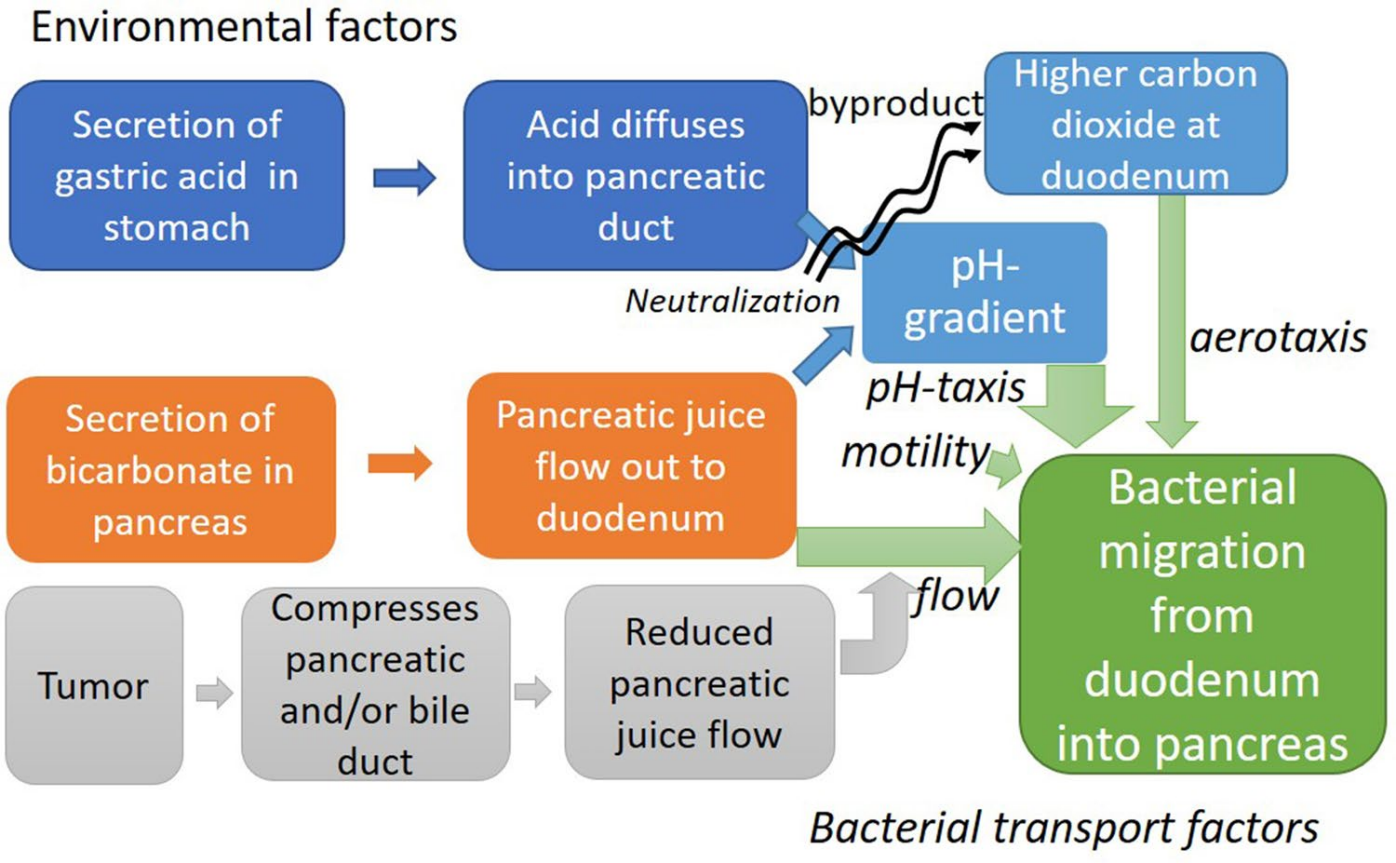
Environmental factors in the upper gastrointestinal tract affect migration of aerobic bacteria from the duodenum into the pancreas via bacterial taxes. The pH-taxis under the pH-gradient between acidic duodenum fluid and pancreatic juice at slightly alkaline pH is the major driving force for migration to pancreas. Aerotaxis away from higher carbon dioxide at the duodenum also increases migration slightly. This migration is counteracted by pancreatic and bile flow but a solid tumor on pancreatic duct at pancreatic head reduces the fluid flow, and thus facilitates migration.

A mechanistic understanding of bacterial migration from the duodenum into the pancreas is provided (Fig. 8). The migration of bacteria into the pancreas in the hepatopancreatic duct seems to depend on a balance between pancreatic juice and bile flow in the duct as convection (this reduces migration) and bacterial pH taxis away from the acidic duodenum toward neutral or slightly alkaline pH in pancreatic juice, more favorable for most bacteria. An imbalance of this (for example, reduced flow in tumor) leads to increased migration. Mathematical modeling predicted bacterial migration into the pancreas with tumors. The pH-tactic behaviors from acidic areas toward neutral pH were validated in a microfluidic study. The mathematical models were further validated by measured upstream migrations of bacteria under flow conditions.

## Mathematical modeling of migration of aerobic bacteria from the duodenum to the pancreas with tumors

Transport of bacteria and oxygen, bicarbonate, carbon dioxide, and hydrogen ion with reactions in the hepatopancreatic duct was mathematically modeled. An anatomical schematic of the upper gastrointestinal tract of pancreas and duodenum modeled is described in Fig. 1. The geometry of the axisymmetric cylindroid was used for hepatopancreatic duct, duodenum walls, and pancreas tissues (Fig. S12). Aerobic bacteria favoring neutral pH, such as *Pseudomonas,* were used as they are typically found bacterial strains in pancreatic cancer^10^ (table S1). A list of the factors included in the modeling is provided in Table 1. The parameter list is provided in table S2. The models are described as follows.

### Migration of aerobic bacteria from the duodenum to the pancreas

Migration of aerobic bacteria from the duodenum to the pancreas is mathematically modeled using a diffusion–advection equation that includes bacterial motility, aerotaxis to oxygen, aerotaxis away from carbon dioxide pH taxis, and pancreatic juice and bile flow (convection), as described in the following governing equation:

5 $$\begin{aligned} & \frac{\partial b}{{\partial t}} = \underbrace {{\mu_{eff} \left( {\frac{{\partial^{2} b}}{{\partial x^{2} }} + \frac{{\partial^{2} b}}{{\partial r^{2} }} + \frac{1}{r}\frac{\partial b}{{\partial r}}} \right)}}_{{{\text{motility}}}}\underbrace {{ - \left\{ {\frac{\partial }{\partial x}\left( {V_{a}^{x} b} \right) + \frac{\partial }{\partial r}\left( {V_{a}^{r} b} \right) + \frac{1}{r}\left( {V_{a}^{r} b} \right)} \right\}}}_{{{\text{aerotaxis}}\;{\text{to}}\;{\text{oxygen}}}}\underbrace {{ - \left\{ {\frac{\partial }{\partial x}\left( {V_{c}^{x} b} \right) + \frac{\partial }{\partial r}\left( {V_{c}^{r} b} \right) + \frac{1}{r}\left( {V_{c}^{r} b} \right)} \right\}}}_{{{\text{aerotaxis}}\;{\text{ away}}\;{\text{ from}}\;{\text{ carbon}}\;{\text{ dioxide}}}} \\ & \quad \underbrace {{ - \left\{ {\frac{\partial }{\partial x}\left( {V_{pH}^{x} b} \right) + \frac{\partial }{\partial r}\left( {V_{pH}^{r} b} \right) + \frac{1}{r}\left( {V_{pH}^{r} b} \right)} \right\}}}_{{\text{pH - taxis}}}\underbrace {{ - \frac{\partial }{\partial x}\left( {v_{h} b} \right)}}_{\substack{ {\text{bile}}\;{\text{and }} \\ {\text{pancreatic }}\;{\text{juice}}\;{\text{ flow}} } } \\ \end{aligned}$$

*b* [CFU ml^–1^] is bacterial concentration, $\mu_{eff}$ [m^2^ s^–1^] is effective random motility coefficient of bacteria, *V*_*a*_ and *V*_*c*_ [m s^–1^] is aerotactic velocity to oxygen and carbon dioxide, respectively, *V*_*pH*_ [m s^–1^] is pH-tactic velocity, and *v*_*h*_ [m s^–1^] is the fluid flow velocity in hepato-pancreatic duct. Superscripts of *x* and *r* indicate the direction of aerotactic and pH taxis. The growth term was not included here, as the period for bacterial migration (less than ten hours) is in general shorter than bacterial growth (> 10 h). Aerobic bacteria that respire only in aerobic conditions with an oxygen substrate with carbon dioxide as a byproduct show aerotaxis to higher oxygen and toward lower carbon dioxide, which were modeled. Chemotactic terms are typically modeled in convective terms in the Keller-Segel model^41^. Note a simplified one-dimensional model of Eq. (4) is provided in supporting information. Each term will be described below in depth.

#### Random motility of bacteria

Random motion of bacteria in the absence of any attractant or repellent is characterized with “run” and “tumble”, where bacteria run straightforward with counter-clockwise rotation of flagellar and tumble to change direction with their clockwise rotation. This random motility of bacteria is *empirically* modeled in diffusion-equation, as in previous literature^51^:

6 $$v_{motility} = - \mu_{eff} \frac{\partial b}{{\partial x}}$$

$\mu_{eff}$[m^2^ s^–1^] is the effective random motility coefficient. The effective random motility coefficient is dependent on the viscosity of the fluid in the hepatopancreatic duct, $\eta_{h}$ [mPa·s], and is described as follows^22^:

7 $$\mu_{eff} = \mu_{0} \left( {\frac{{\eta_{w} }}{{\eta_{h} }}} \right)^{2}$$

$\eta_{w}$ [mPa·s] is the viscosity of water. Therefore, the viscosity in the hepatopancreatic duct should be lower than that in bile or pancreatic juice as they are diluted there, as calculated using the viscosity of pancreatic juice, $\eta_{p}$ [mPa·s], pancreatic juice flow, *U*_*p*_ [ml min^–1^] and bile flow rate, *Q*_*b*_ [ml min^–1^] as:

8 $$\eta_{h} = \frac{{Q_{p} }}{{Q_{p} + Q_{b} }}\eta_{p}$$

This is based on the assumption that pancreatic juice and bile acid contribute to viscosity independently. This is justified as pancreatic juice viscosity is due to enzymatic proteins, while the viscous contrition of bile is due to bile acids. Using parameters of viscosity of pancreatic juice of 1.5 mPa·s^52^, bile flow rate at 0.43 ml min^–1^, and pancreatic juice flow rate at 0.2 ml min^–1^ (table S2), the viscosity due to pancreatic juice is at 0.95 mPa·s (Eq. 4). The viscosity of bile at 0.90 mPa·s^53^ is lower than that (0.95 mPa·s). Thus, a viscosity of 0.95 mPa·s is used for that in the hepatopancreatic duct. Pancreatic tissues are considered porous media, and the random motility coefficient in pancreatic tissues is described using tortuosity $\tau$ [ −] and porosity $\phi$ [ −] as follows:

9 $$D_{eff}^{{O_{2} }} = D_{0}^{{O_{2} }} \frac{{\eta_{w} }}{{\eta_{h} }} \cdot \frac{\phi }{\tau }{ (}0 \, < \, r < r_{h} {)}$$

Bacterial transport across the wall of the duct is described using permeability of the duct of bacteria, *P*_*b*_ [m s^–1^] as follows:

10 $$Flux_{b} \left( {r = r_{h} } \right) = P_{b} \left\{ {b\left( {r = r_{h} } \right)_{wall}^{{}} - b\left( {r = r_{h} } \right)_{duct}^{{}} } \right\}$$

where *b*(*r* = *r*_*h*_)_*wall*_ and *b*(*r* = r_h_)_*duct*_ are bacterial concentrations on the ductal wall in the duodenum wall and hepatopancreatic duct, respectively*. r*_*h*_ [mm] is the radius of the hepato-pancreatic duct. Note the unit of flux is CFU m^–2^ s^–1^. The permeability of the bile duct for bacteria was determined from measurements in rats in the literature. The permeability of the human bile duct is estimated using a bile duct wall thickness of 80 μm in mice^54^ and that in humans at 0.5 mm^55^.

#### Aerotaxis

Bacteria monitor their cellular energy levels and respond to a decrease in energy by swimming to a microenvironment that reenergizes the cells^56,57^. Thus, bacteria migrate toward optimal oxygen and carbon dioxide levels for better energy production by using a strategy called "energy taxis"^52^. Additionally, carbon dioxide also works as a repellent for aerobes. In aerotaxis, bacteria use sensing mechanisms called 'logarithmic sensing,' where bacteria sense the logarithm of the concentration gradient^58^. A modified Keller-Segel model, Lapidus and Schiller model^59^, is used for logarithmic sensing of the aerotactic term for oxygen in Eq. (11):

11 $$V_{a}^{x} = \chi_{0}^{a} \frac{{K_{d} }}{{\left( {K_{d} + a} \right)^{2} }} \cdot \frac{\partial a}{{\partial x}}$$

where *a* [mol l^–1^] is the oxygen concentration, $\chi_{0}^{a}$ [m^2^ s^–1^] is the chemotactic sensitivity coefficient of bacterial aerotaxis, and *K*_*d*_ [mmol l^–1^] is the dissociation constant. Note that aerotactic velocity is independent of viscosity^60^. Aerotaxis away from higher carbon dioxide is described in:

12 $$V_{c}^{x} = - \chi_{0}^{c} \frac{1}{{\left( {K_{d}^{c} + c} \right)}} \cdot \frac{\partial c}{{\partial x}}$$

*K*_*d*_^*c*^ [mmol/] is the dissociation constant for the ligand and receptor for carbon dioxide. A typical chemotactic sensitivity coefficient of 1 × 10^–8^ m^2^ s^–1^ is used.

#### pH-taxis

Bacteria that grow optimally in a pH range of near neutral require robust mechanisms for cytoplasmic pH homeostasis to survive and, in some cases, grow during exposure to acidic or alkaline conditions that are well outside the pH range tolerated for cytoplasmic pH^60–64^. A sensing mechanism is called 'pH taxis’, a bidirectional behavior that migrates away from extremely acidic and alkaline environments and to optimal pH. A continuum-based mathematical model for bacterial pH taxis is developed here based on a traditional chemotaxis Keller-Segel (K-S) model. Chemotactic velocity, *V*_*c*_ [m/s], is proportional to the logarithm of the chemoattractant (or chemorepellent) concentration gradient, as described in $V_{c} = {\chi \mathord{\left/ {\vphantom {\chi c}} \right. \kern-\nulldelimiterspace} c} \cdot {{\partial c} \mathord{\left/ {\vphantom {{\partial c} {\partial x}}} \right. \kern-\nulldelimiterspace} {\partial x}}$, where *c* [mol/l] is the chemoattractant or chemorepellent concentration and $\chi$ [m^2^ s^–1^] is the chemotactic sensitivity coefficient. However, this equation cannot be applied to pH taxis, as pH-tactic bacteria exhibit bidirectional behavior, i.e., away from alkaline and acidic pH toward neutral pH. Therefore, we modified the K-S model so that bacteria can sense the logarithm of "differences of concentration from optimal concentration”, as described in the following:

13 $$V_{pH}^{x} = \chi_{0}^{pH} \frac{{d\left( {\ln ([H^{ + } ] - [H^{ + } ]_{0} )} \right)}}{dx} = \chi_{0}^{pH} \frac{1}{{\left( {[H^{ + } ] - [H^{ + } ]_{0} } \right)}} \cdot \frac{{d\left( {[H^{ + } ]} \right)}}{dx}$$

where *[H*^+^*]*_*0*_ is the optimal hydrogen ion concentration for bacteria, *[H*^+^*]* is the hydrogen ion concentration, and $\chi_{0}^{pH}$ [m^2^ s^–1^] is the pH-tactic sensitivity coefficient. This model was validated using literature data by Zheng and coworkers with their permissions^63^. The details of validation are provided in supplemental materials (Fig. S13).

#### Ion equilibrium and transport

Pancreatic juice contains bicarbonate, *HCO*_*3*_^*–*^_,_ at approximately 80 mmol l^–1^ in the fasting state after the stomach is emptied^65^, and this bicarbonate neutralizes gastric acid in the duodenum in the following equation:

14 $$H^{ + } + HCO_{3}^{ - } \underset{k - }{\overset{k + }{\longleftrightarrow}}H_{2} O + CO_{2}$$

Equilibrium equation in Eq. (14) is described using dissociation constants *K*_*1*_ [mol l^–1^] and *K*_*2*_ [-]:

15 $$\frac{{[H^{ + } ][HCO_{3}^{ - } ]}}{{pCO_{2} }} = \frac{{k_{ - } }}{{k_{ + } }} = K_{1} K_{2} = K^{*}$$

K^*^ = 10^–6.1^ mol l^–1^ and *k*_*-*_ = 3.71 × 10^–2^ s^–1^ from the literature^64^.

Details of mathematical models of transport and equilibrium of ions and oxygen in the duct are provided in the supplemental materials.

#### Fluid flow velocity in the hepato-pancreatic duct

The bile duct and pancreatic duct joints together at the distal pancreas, consisting of a hepatopancreatic duct or common channel 1–11 mm in length^66,67^, open in the duodenum. Thus, fluid flow in the hepatopancreatic duct is caused by both bile and pancreatic juice. Bile and pancreatic juice flow rates were calculated from daily total bile flow at 620 ml day^–1^ (0.43 ml min^–1^)^68,69^, and the pancreatic juice flow rate during the fasted state, after gastric emptying after meal and secretion rate is lower, was 0.2–0.3 ml min^–1^. The flow rate of a *fasted* period, after meal and gastric emptying, is used here, as migration should be more straightforward during this period, when bile and pancreatic juice secretions are lower^70^. The volumetric flow rate in the hepatopancreatic duct, *Q*_*h*_ [ml/min], is thus calculated as follows:

16 $$Q_{h} = Q_{p} + Q_{b}$$

The Reynolds number in the hepatopancreatic duct was calculated using the following equation:

17 $${\text{Re}} = \frac{{2\rho r_{h} Q_{h} }}{{\eta_{h} A}}$$

$\sigma$ [g/m^3^] is density of the fluid (assumed 1 kg/m^3^]. The volumetric flow rate in the hepatopancreatic duct of healthy individuals due to both bile and pancreatic juice, *Q*_*h*_ [m^3^ s^–1^], is 0.63 ml min^-1^. $\eta$ mPa·s [g m^–1^ s^–1^] is viscosity and *A* [m^2^] is the cross-sectional area of the duct, The Reynolds number in the hepatopancreatic duct was calculated to be 0.0017, assuring laminar flow. Thus, the fluid velocities in the duct at the ductal radius of *r* [mm], *v*_*h*_*(r),* follow the Hagen-Poiseuille law as:

18 $$v_{h} \left( r \right) = v_{\max } \left\{ {1 - \left( {\frac{{r^{{}} }}{{r_{h}^{{}} }}} \right)^{2} } \right\}$$

*r*_*h*_ [mm] is the radius of the hepatopancreatic duct. The maximum flow velocity, *v*_*max*_ [m s^–1^], is calculated as:

19 $$v_{\max } = \frac{{2Q_{h} }}{{\pi r_{h}^{2} }}$$

The maximum flow velocity in the hepatopancreatic duct for a *healthy* individual is 494 μm s^–1^. The pancreatic juice flow rate of pancreatic cancer patients is 1/4 that of healthy individuals in the literature^30^. The flow rate of bile for cancer patients is missing in the literature. Therefore, the flow rate of patients with obstruction due to bile stones at 56–373 ml/day (212 ml/day on average)^69^ is used in the models. The maximum flow rate in the duct for cancer patients is calculated at 126 μm s^–1^.

### Boundary conditions and numerical simulations

The governing equations were numerically solved using COMSOL Multiphysics 5.0 with initial and boundary conditions below. The bacterial concentration in duodenum fluid at 10^4^ CFU ml^–1^ was used for the boundary condition^71^:

20 $$b\left( {x = 0} \right) = 10^{4} {\text{ CFU ml}}^{{ - 1}}$$

The oxygen concentration in the human duodenum is not available in the literature. The oxygen concentration in the stomach is 58 mmHg in mice, while that in the duodenum is 32 mmHg^72^ Oxygen level in the human stomach is at 15–16%^72,73,74^. Using this ratio of oxygen concentration in mice and equilibrium the oxygen concentration to air at 37 °C at 0.21 mmol l^–1^, oxygen concentration in duodenum at 0.083 mmol l^–1^ is used. Oxygen concentration in tumors at 15 mmHg is also used^74^

21 $$a\left( {x = 0} \right) = 0.083{\text{ mmol l}}^{{ - 1}}$$

22 $$a\left( {x = x_{d} } \right) = 0.039{\text{ mmol l}}^{{ - 1}}$$

In the preliminary simulation studies, the distance between the duodenum and pancreatic tumor did not affect the oxygen concentration gradient between the duodenum and the pancreatic tumor. The pH of fasted human duodenum at 4.9 is used^38^

23 $$pH(x = 0) = 4.9$$

An initial carbon dioxide concentration of 5% (2.64 mmol/l) was used.

24 $$[CO_{2} ]_{0} (t = 0) = {2}{\text{.64 mmol/l}}$$

The bicarbonate concentration in pancreatic juice during the fasting period is 80 mmol l^–1^.

25 $$[HCO_{3}^{ - } ]_{0} (t = 0) = 80{\text{ mmol/l}}$$

Bacterial concentration in pancreas at eight hours, which is the longest duration of fasting period after meal, when the stomach is emptied, were calculated.

## Experimental methods

### Bacterial pH taxis in a microfluidic device

A polydimethylsiloxane (PDMS) microfluidic device that can generate a steady concentration gradient with double-layered flow was fabricated. PDMS elastomer base (SILPOT™ 184 Silicone Elastomer Base) was mixed with a curing agent (SILPOT™ 184 Silicone Elastomer Curing Agent) at a ratio of 10:1. The PDMS mixture was degassed using a vacuum chamber (G-20DA, ULVAC KIKO. Inc., Japan). This was poured onto the metal mold, designed for the device and created previously (Fig. S14), and cured by heating at 75 °C for two hours. PDMS was peeled off of the metal mold. Both surfaces of the PDMS microfluidic device and a sliding glass were irradiated with oxygen plasma (SEDE-P, meiwafosis, Japan) at 10 pascals at 5 mA for 35 s. Both were attached to each other and heated at 90 °C for one hour to permanently bond.

### Preparation of bacteria

GFP *E. coli* (ATCC 25922™) were purchased from the American Type Culture Collection (ATCC) and recovered following them. GFP *E. coli* (ATCC 25922™) clone, which was derived from ATCC® 25922™, contains a multicopy vector encoding the green fluorescent protein GFPmut3. This gene is expressed under the control of the Plac promoter. *E. coli* were chosen here as the objective this experiment is to obtain basic data for bacterial pH-taxis. Bacteria were cultured in LB broth with stirring using a magnetic stirrer at 37 °C at least overnight. The obtained bacterial culture at exponential phase was centrifuged at 4000 rpm for ten minutes. The bacterial pellet was then washed in distilled water and centrifuged again. The pellet was then diluted into hydrochloride or bicarbonate solution.

Syringe pumps (Aladdin 1000, US) were connected to the microfluidic device. Bicarbonate (80 mmol/l) and hydrochloride (10^–3^ mol/l) solutions were poured at 200 μl/min from inlets 1 and 2, respectively (Fig. 6a). *GFP E. coli* were included in either of them. Bacterial distribution was measured from the fluorescence of *GFP E. coli* under UV light (350 nm) using a digital single lens reflex (D5100, Nikon, Japan) in black-and-white mode. The pH in the microfluidic channel was visualized using bromothymol blue solution (Fig. 6a) or phenolphthalein solution (Sigma Aldrich, Japan) (figure S6). The obtained images were analyzed using ImageJ (NIH, US). The relative brightness was calculated as (*G*_*max*_ − *G*)(*G*_*max*_ − *G*_*min*_).

### Upstream swimming of bacteria in different pH solutions against flow

Upstream migration of *Pseudomonas fluorescens* (ATCC 13525) from hydrochloride solution or sodium bicarbonate against bicarbonate solution flow was analyzed using a T-shaped cylinder fabricated by referring to previous literature^75^. Details are followed. First, the degassed mixture of PDMS was poured into a 50-mm diameter petri dish with a thickness of a few millimeters as a basis for the cylinder (figure S16a). This PDMS mixture was cured at 75 °C for two hours. Then, glass tubes were placed in T-shaped tubes, and another PDMS mixture was poured there (Fig. S16b, c). The tubes were then removed carefully by incising with a cutter, leaving a hollow T-shaped cylinder (figure S16d). End tips of the hollowed cylinders were filled with remaining cured PDMS so that the PDMS that would be poured later would not be filled in. Finally, the PDMS mixture was poured into the whole device and cured (figure S16e).

*Pseudomonas fluorescens* (ATCC 13525) was chosen here because *Pseudomonas* is one of the most common strains in pancreatic cancer^10^, and they can be seen using their intrinsic fluorescence with UV excitation and emission at 340 nm. Five-milliliter syringes of hydrochloride (approximately 10^–4.9^ mol/l) or sodium bicarbonate (80 mmol/l) solution containing bacteria were connected to the upper inlet of the T-shaped cylinder. Bacteria in hydrochloride solution were prepared by diluting the bacterial pellet obtained by centrifugation with hydrochloride at the desired concentration. The pH was adjusted by the color of bromocresol purple (Wako Chem., Japan). This concentration of hydrochloride is chosen because that of fasted duodenum is at 4.9–5.5^38^. The flow rates were 200 μl/min and 20 μl/min. The pH distribution was measured by bromocresol purple (FujifilmWako, Japan). Bacteria were measured using a CMOS image sensor (IMX586, Sony, Japan) under 350 nm light. The obtained movies were analyzed using MATLAB 2021 (MathWorks, Japan), as shown in Fig. 7e. The G values in the movies at the T-junction were extracted. Then the average G values in the vertical sections were calculated. Thus G-value distributions along the horizontal axis, corresponding to the penetration depth, where bacterial concentrations changes in the right cylinder containing bicarbonate with flow, were obtained for each frame (Fig. 7e). Note horizontal distance in millimeters was calculated from a ruler in an image placed near the device.

## Supplementary Information

Supplementary Video 1.

Supplementary Video 2.

Supplementary Video 3.

Supplementary Video 4.

Supplementary Information 1.

Supplementary Information 2.

## References

[1] Rawla P, Sunkara T, Gaduputi V (2019). Epidemiology of pancreatic cancer: global trends, etiology and risk factors. World J Oncol..

[2] Gopalakrishnan V (2018). Gut microbiome modulates response to anti-PD-1 immunotherapy in melanoma patients. Science.

[3] Gaiser RA (2019). Enrichment of oral microbiota in early cystic precursors to invasive pancreatic cancer. Gut.

[4] Li, S., G.M. Fuhler, N. BN, T. Jose, M.J. Bruno, M.P. Peppelenbosch, and S.R. Konstantinov. (2017). Pancreatic cyst fluid harbors a unique microbiome. *Microbiome*. 5. 10.1186/s40168-017-0363-6 PMC5680603 29122007

[5] Lillioja S, Mott DM, Spraul M, Ferraro R, Foley JE, R.E. et al. (1993). *The New England Journal of Medicine*. Massachusetts Medical Society. All rights reserved. N. Engl. J. Med. 29: 1230–5.

[6] Schmid SW, Uhl W, Friess H, Malfertheiner P, Büchler MW (1999). The role of infection in acute pancreatitis. Gut.

[7] Thomas RM (2018). Intestinal microbiota enhances pancreatic carcinogenesis in preclinical models. Carcinogenesis.

[8] Guo W, Y. (2021). Tumor microbiome contributes to an aggressive phenotype in the basal-like subtype of pancreatic cancer. Commun. Biol..

[9] Nejman D (2020). The human tumor microbiome is composed of tumor type-specific intracellular bacteria. Science.

[10] Geller LT (2017). Potential role of intratumor bacteria in mediating tumor resistance to the chemotherapeutic drug gemcitabine. Science.

[11] Thomas RM, Jobin C (2020). Microbiota in pancreatic health and disease: the next frontier in microbiome research. Nat Rev Gastroenterol Hepatol.

[12] Pushalkar S (2018). The pancreatic cancer microbiome promotes oncogenesis by induction of innate and adaptive immune suppression. Cancer Discov.

[13] Riquelme E (2019). Tumor microbiome diversity and composition influence pancreatic cancer outcomes. Cell.

[14] Chandra V, McAllister F (2021). Therapeutic potential of microbial modulation in pancreatic cancer. Gut.

[15] Ezenobi NO, Okpokwasili GC (2016). Combined effect of temperature and pH on *Pseudomonas aeruginosa* isolated from a cosmetic product. Int. J. Curr. Res..

[16] Gunasekaran V, Kotay SM, Das D (2006). Alkaline lipase production by Citrobacter freundii IIT-BT L139. Indian J Exp Biol.

[17] Abbas SZ (2014). Isolation and characterization of arsenic resistant bacteria from wastewater. Braz J Microbiol.

[18] Beal C, Louvet P, Corrieu G (1989). Influence of controlled pH and temperature on the growth and acidification of pure cultures of Streptococcus thermophilus 404 and Lactobacillus bulgaricus 398. Appl Microbiol Biotechnol.

[19] Del Castillo E (2019). The microbiomes of pancreatic and duodenum tissue overlap and are highly subject specific but differ between pancreatic cancer and noncancer subjects. Cancer Epidemiol Biomarkers Prev.

[20] Sung JY, Costerton JW, Shaffer EA (1992). Defense system in the biliary tract against bacterial infection. Dig Dis Sci.

[21] Ranjbaran, M., and A.K. Datta. (2021). Engineering modeling frameworks for microbial food safety at various scales.: 1–37. 10.1111/1541-4337.12818 34486219

[22] Shirai H, Datta AK, Oshita S (2017). Penetration of aerobic bacteria into meat : a mechanistic understanding. J Food Eng.

[23] Ranjbaran, M., and A.K. Datta. (2019). Retention and infiltration of bacteria on a plant leaf driven by surface water evaporation. *Phys. Fluids.* 31: 112106.

[24] Figueroa-Morales N, Dominguez-Rubio L, Ott TL, Aranson IS (2019). Mechanical shear controls bacterial penetration in mucus. Sci Rep.

[25] Kaya T, Koser H (2012). Direct upstream motility in Escherichia coli. Biophys J.

[26] Marcos HC, Fu TRP, Stocker R (2012). Bacterial rheotaxis. Proc. Natl. Acad. Sci. U. S. A..

[27] Figueroa-Morales N, et al. (2020) *E. coli* “supercontaminates” narrow ducts fostered by broad run-time distribution. *Sci Adv* 6(11):1–8. 10.1126/sciadv.aay0155 PMC7069694 32201716

[28] Diao J (2006). A three-channel microfluidic device for generating static linear gradients and its application to the quantitative analysis of bacterial chemotaxis. Lab Chip.

[29] Rinderknecht H, Renner IG, Stace NH (1983). Abnormalities in pancreatic secretory profiles of patients with cancer of the pancreas. Dig Dis Sci.

[30] Carr-Locke DL (1980). Serum and pancreatic juice carcinoembryonic antigen in pancreatic and biliary disease. Gut.

[31] Gregg JA, Sharma MM (1978). Endoscopic measurement of pancreatic juice secretory flow rates and pancreatic secretory pressures after secretin administration in human controls and in patients with acute relapsing pancreatitis, chronic pancreatitis, and pancreatic cancer. Am J Surg.

[32] Kruse EJ (2010). Palliation in pancreatic cancer. Surg Clin North Am..

[33] Muir CA (2004). Acute ascending cholangitis. Clin J Oncol Nurs.

[34] Mitchell JG, Kogure K (2006). Bacterial motility: Links to the environment and a driving force for microbial physics. FEMS Microbiol. Ecol..

[35] Boyd A, Simon M (1982). Bacterial chemotaxis. Annu. Rev. Physiol..

[36] Hu B, Tu Y (2013). Precision sensing by two opposing gradient sensors: How does escherichia coli find its preferred pH level?. Biophys. J..

[37] Taylor BL, Zhulin IB, Johnson MS (1999). Aerotaxis and Other Energy-Sensing Behavior in Bacteria. Annu. Rev. Microbiol..

[38] Ovesen L (1986). Intraluminal pH in the stomach, duodenum, and proximal jejunum in normal subjects and patients with exocrine pancreatic insufficiency. Gastroenterology.

[39] Guyton AC. Human physiology and mechanism of disease. 1987: 499.

[40] Malin, G., and Walsby, A. E. Chemotaxis of a cyanobacterium on concentration gradients of carbon dioxide, bicarbonate and oxygen. *J Gerrral Microbiol* **131**, 2643–3265 (1985).

[41] Keller EF, Segel LA (1971). Model for chemotaxis. J Theor Biol.

[42] Johns Hopkins Medicine. Sphincter of Oddi Dysfunction. https://www.hopkinsmedicine.org/gastroenterology_hepatology/_pdfs/pancreas_biliary_tract/sphincter_of_oddi_dysfunction.pdf. Accessed 17 Dec 2021.

[43] Abdeldayem H, Ghoneim E, Refaei AA-E, Abou-Gabal A (2007). Obstructive jaundice promotes intestinal-barrier dysfunction and bacterial translocation: experimental study. Hepatol. Int..

[44] Bhattacharjee, T., and S.S. Datta. (2019). Bacterial hopping and trapping in porous media. Nat. Commun. 10. 10.1038/s41467-019-10115-1 PMC6502825 31061418

[45] Westphal K, Leschner S, Jablonska J, Loessner H, Weiss S (2008). Containment of tumor-colonizing bacteria by host neutrophils. Cancer Res..

[46] Fan X (2018). Human oral microbiome and prospective risk for pancreatic cancer: a population-based nested case-control study. Gut.

[47] Bovo PG (1995). Intraluminal gastric pH in chronic pancreatitis. Gut..

[48] Nakano S, et al. (2020) Association between the use of antibiotics and efficacy of gemcitabine plus nab-paclitaxel in advanced pancreatic cancer. *Medicine (Baltimore)* 10.1097/MD.0000000000022250 PMC7523777 32991420

[49] Imai H (2019). Antibiotic therapy augments the efficacy of gemcitabine-containing regimens for advanced cancer: a retrospective study. Cancer Manag Res.

[50] National Library of Medicine (U.S.). (2005, March - Fecal Microbial Transplants for the Treatment of Pancreatic Cancer. Identifier NCT04975217.

[51] Ford RM, Lauffenburger DA (1991). Measurement of bacterial random motility and chemotaxis coefficients: II: application of single-cell-based mathematical model. Biotechnol. Bioeng..

[52] Harada H, Ueda O, Kochi F, Kobayashi T, Komazawa M (1981). Comparative studies on viscosity and concentration of protein and hexosamine in pure pancreatic juice. Gastroenterol Jpn..

[53] Reinhart WH, Näf G, Werth B (2010). Viscosity of human bile sampled from the common bile duct. Clin Hemorheol Microcirc.

[54] Smith D, Boyer L (1982) Permeability characteristics of bile duct in the rat. Am J Physiol.;242(1):G52–7. 10.1152/ajpgi.1982.242.1.G52 7058899

[55] Testoni PA (2006). Main pancreatic duct, common bile duct and sphincter of Oddi structure visualized by optical coherence tomography: an ex vivo study compared with histology. Dig Liver Dis.

[56] Taylor BL, Zhulin IB, Johnson MS (1999). Aerotaxis and other energy-sensing behavior in bacteria. Annu Rev Microbiol.

[57] Taylor BL (1983). How do bacteria find the optimal concentration of oxygen?. Trends Biochem Sci.

[58] Menolascina F (2017). Logarithmic sensing in Bacillus subtilis aerotaxis. Npj Syst Biol Appl.

[59] Lapidus IR, Schiller R (1976). Model for the chemotactic response of a bacterial population. Biophys J.

[60] Schauer O, et al. (2018) Motility and chemotaxis of bacteria-driven microswimmers fabricated using antigen 43-mediated biotin display. *Sci Rep* 8(1):1–11. 10.1038/s41598-018-28102-9 PMC6023875 29955099

[61] Sachs G, Padan E (2011). Molecular aspects of bacterial pH sensing and homeostasis. Nat Rev Microbiol.

[62] Yang Y, Sourjik V (2012). Opposite responses by different chemoreceptors set a tunable preference point in Escherichia coli pH taxis. Mol Microbiol.

[63] Zhuang J, Carlsen RW, Sitti M (2015) PH-taxis of biohybrid microsystems. *Sci Rep* 5:1–13. 10.1038/srep11403 PMC4466791 26073316

[64] Hu B, Tu Y (2014) Behaviors and Strategies of Bacterial Navigation in Chemical and Nonchemical Gradients. *PLoS Comput Biol* 10(6):e1003672. 10.1371/journal.pcbi.1003672 PMC4063634 24945282

[65] Gerolami A (1989). Calcium carbonate saturation in human pancreatic juice: possible role of Ductal H+ secretion. Gastroenterology.

[66] Higashiyama H (2016). Anatomy of the murine hepatobiliary system: a whole-organ-level analysis using a transparency method. Anat Rec.

[67] Higashiyama H (2018). Anatomy and development of the extrahepatic biliary system in mouse and rat: a perspective on the evolutionary loss of the gallbladder. J Anat.

[68] Esteller A (2008). Physiology of bile secretion. World J Gastroenterol.

[69] Ohya, T., Murakami, E., Kougame, A., Numata, Y., and Hiraoka, M. (2013). Proposal of endoscopic lithotripsy for common bile duct stones based on the pathogenesis of stone formation. C*holithiasis. Ann Surg* 238(1), 97–102 in Japanese.

[70] Glasbrenner B, Dürrschnabel L, Büchler M, Malfertheiner P (1992). Nonparallel patterns of circadian pancreatic and biliary secretions in fasting rats. Int J Pancreatol.

[71] Sender R, Fuchs S, Milo R (2016). Revised estimates for the number of human and bacteria cells in the body. PLoS Biol.

[72] He G (1999). Noninvasive measurement of anatomic structure and intraluminal oxygenation in the gastrointestinal tract of living mice with spatial and spectral EPR imaging. Proc Natl Acad Sci USA.

[73] Matsumoto S (2018). Metabolic and physiologic imaging biomarkers of the tumor microenvironment predict treatment outcome with radiation or a hypoxia-activated prodrug in mice. Cancer Res.

[74] Britannica Health & Medicine, Anatomy & Physiology Intestinal gas. https://www.britannica.com/science/intestinal-gas. Accessed Dec.18th, 2020.

[75] Pauty J (2018). A vascular endothelial growth factor-dependent sprouting angiogenesis assay based on an in vitro human blood vessel model for the study of anti-angiogenic drugs. EBioMedicine.

