# Supplementary figures and images for: pH-taxis drives aerobic bacteria in duodenum to migrate into the pancreas with tumors
Source: Sci Rep. 2022 Feb 2;12:1783. doi: 10.1038/s41598-022-05554-8 (PMC8810860; doi:10.1038/s41598-022-05554-8)

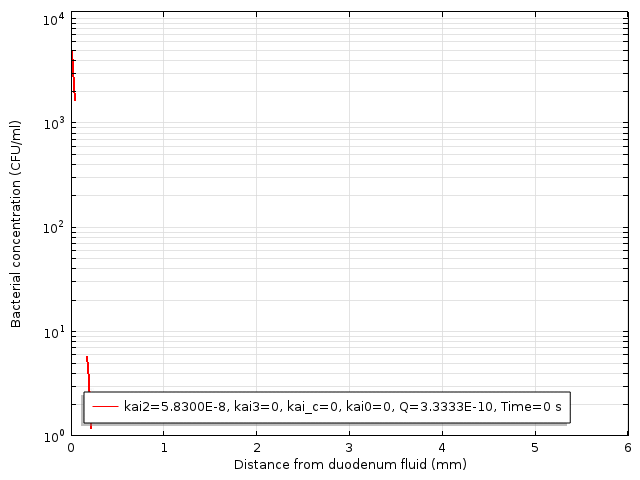

Supplement: Supplementary file 3 — Supplementary Video 3. [file 41598_2022_5554_MOESM3_ESM.gif]

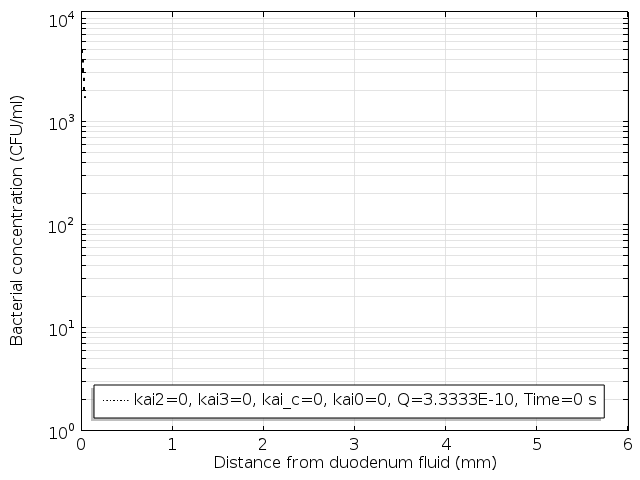

Supplement: Supplementary file 4 — Supplementary Video 4. [file 41598_2022_5554_MOESM4_ESM.gif]
