# Supplementary material for: pH-taxis drives aerobic bacteria in duodenum to migrate into the pancreas with tumors
Source: Sci Rep. 2022 Feb 2;12:1783. doi: 10.1038/s41598-022-05554-8 (PMC8810860; doi:10.1038/s41598-022-05554-8)
Supplement: Supplementary file 5 — Supplementary Information 1. [file 41598_2022_5554_MOESM5_ESM.pdf]

Supporting information

pH-taxis drives aerobic bacteria in duodenum to migrate into the pancreas with tumors

Hiroaki Shirai[a*] Cocoro Ito[b], Kosuke Tsukada[a,b]

Affiliations:

[a]Graduates School of Science and Technology, Keio University, 3-14-1 Hiyoshi Kohoku-ku, Yokohama-shi, Kanagawa, Japan 223-8522

[b]Faculty of Science and Technology, Keio University

.

Table S1. Bacterial strains found in pancreatic cancer in previous literature

Bacterial strain	Percentage read (Geller et al., 2017)(1)	Relative abundance (LTS/STS)(Riquelme et al., 2019)(2)	Aerobic or anaerobe	Motility	Optimal pH
Geller et al. (2017) (bacteria in pancreatic tumors)					
Pseudomonas putida	11.8%	-	Aerobic	Motile(3)	8(4)
Citrobacter freundii	8.9%	-	Aerobic	Motile	8–9(5)
Klebsiella pneumoniae	8.9%	-	Facultative anaerobe	*1	6–8(6)
Enterococcus faecalis	3.5%	-	Facultative anaerobe	Im-motile	
Riquelme et al. (2019) (bacteria in pancreatic tumors)					
Bacillus clausii	-	1.8%/0%	Aerobic	Motile	
Saccharopolyspora rectivirgula	-	1.9%/0.26%	Aerobic	Non-motile	

Staphylococcus cohnii	-	1.3%/0.07%	Aerobic	Non-motile	
Pseudoxanthomonas taiwanensis	-	0.94%/0.0048%	Aerobic		
Weissella paramesenteroides	-	0.77%/0.0024%		Motile	
Acinetobacter bouveti	-	1.6%/0%	Aerobic	(twitting) motility(7)	
Erwinia mallotivora	-	4.5%/0.0024%	Aerobic (facultative aerobe)	Motile	
Dietzia alimentaria		3.8%/6.7%	Aerobic	Non-motile[*2]	
Guo et al. (2021) (bacteria in pancreatic tumors)					
Pseudomonas			Aerobic	motile	8
Elizabethkingia			Aerobic	Non-motile	
Gaiser et al. (2019) (Oral bacteria in pancreatic cystic fluid)					

Fusobacterium nucleatum			Obligate anaerobe	Im-motile<br>gliding motility	7.4(8)
Granulicatella adiacens			Facultative anaerobe	Non-motile	

Strains in Riquelme et al. (2019) are in a descending order in Mann-Whitney Test. * 1: typically considered im-motile but evidence for motile and flagellar were shown (9)(10). *2: chosen as it was highly abundant strain in both LTS and STS. Bacteria with motility and neutral optimal pH exhibit pH-taxis.

Table S2. Full parameter list used for the modeling

Symbol	Definition	Value	literature
a	Oxygen concentration	Eqn. (20)	
a_d	Oxygen concentration in duodenum fluid	0.083 mmol l^{-1}	(11)
b	Bacterial concentration	Eqn. (1)	
b_d	Bacterial concentration in duodenum fluid	10^4 CFU ml^{-1}	(12)
d_b	Diameter of common bile duct	4.1 mm	(13)
d_p	Diameter of pancreatic duct	3 mm	(14)
d_w	Thickness of duodenal wall	1.6 mm	(15)
w_p	Thickness of pancreatic duct wall	0.47 mm	(16)
w_b	Thickness of common bile duct wall	0.46 mm	(17)
D_0^{H+}	Diffusion coefficient of hydrogen ion in water	9.3 x 10^{-9} m^2 s^{-1}	(18)
D_0^{O2}	Diffusion coefficient of oxygen in water at 37°C	3.2 x 10^{-10} m^2 s^{-1}	(19)
D_{wall}	Diffusion coefficient of oxygen in small intestinal wall	0.1 mm^2 s^{-1}	(20)
k_+	Rate constant for reaction of bicarbonate and hydrogen ion to carbon dioxide	Eqn. (12)	
k_-	Rate constant for reaction of carbon dioxide to bicarbonate and hydrogen ion	3.71 x 10^{-2} s^{-1}	(21)

η

K^*	Dissociation constant of carbon dioxide, bicarbonate and hydrogen ion	$10^{-6.1}$ mol l^{-1}	
K_d	Chemoreceptor coefficient for bacterial aerotaxis	13.9 μmol l^{-1}	(22)
P_b	Permeability of hepato-pancreatic duct of bacteria	4.1 x 10^{-7} m s^{-1}	See text
P_{O2}	permeability of oxygen	2.8 x 10^{-6} m s^{-1}	See text
Q_b	Bile flow rate of healthy individuals	620 ml day^{-1} (0.4 ml min^{-1})	(23)
Q_p	Pancreatic juice flow rate of human during fasting period	0.2 ml min^{-1}	(24)
Q_b^c	Bile flow rate of pancreatic cancer patients*	0.15 ml min^{-1} (211 ml day^{-1})*	(25)
Q_p^c	Pancreatic juice flow rate of pancreatic cancer patients	0.05 ml min^{-1}	(26)
V_{pH}	pH-tactic velocity	Eqn. (9)	
v	Fluid velocity in hepato-pancreatic duct	Eqn. (24)	
v_{max}	Maximum fluid velocity in hepato-pancreatic duct	Eqn. (25)	
η_p	Viscosity of pancreatic juice	1.4 mPa·s	(27)
η_b	Viscosity of bile duct	0.94 mPa·s	(28)

η_w	Viscosity of water at 37°C	0.691 mPa·s	(29)
μ_0	Random motility coefficient of bacteria in water	1 x 10^{-9} m^2 s^{-1}	
τ	Tortuosity of pancreatic tissues	1.4	
ϕ	Porosity of pancreatic tissues	0.26	
χ_0	Chemotactic sensitivity coefficient for bacterial aerotaxis	5.79 x10^{-8} m^2 s^{-1}	
χ_0	Chemotactic sensitivity coefficient for bacterial pH-taxis less than optimal pH	5.83 x10^{-8} m^2 s^{-1}	(30)
$[H]$	Hydrogen ion concentration	Eqn. (19)	
$[HCO_3^-]$	Bicarbonate concentration	Eqn. (20)	
$[HCO_3^-]_0$	Bicarbonate concentration in pancreatic juice of fasted pancreas	80 mmol l^{-1}	(31)
$[CO_2]$	Carbon dioxide concentration	Eqn. (21)	
$[CO_2]_0$	Carbon dioxide concentration in pancreatic juice	1.6 mmol/l	(32)

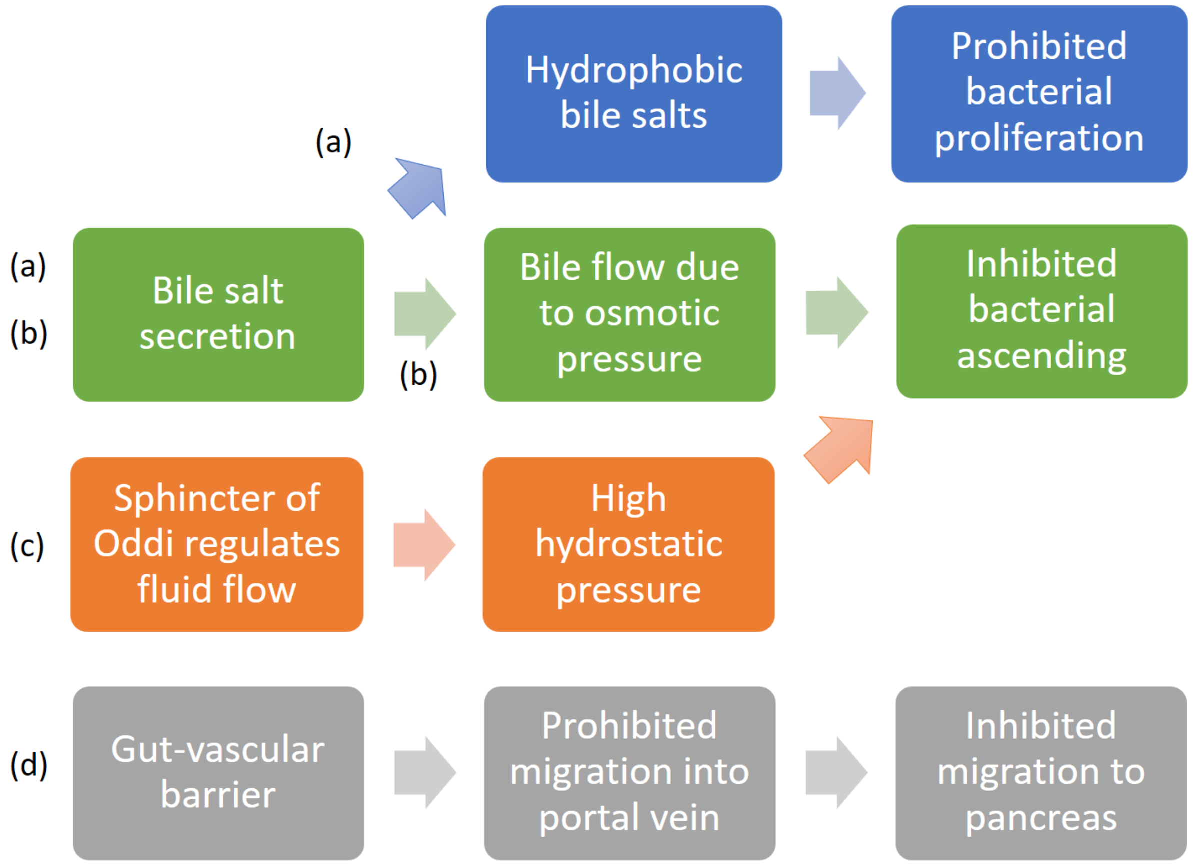

Figure S1. Defense mechanisms against bacterial invasion from the intestine into the pancreas. Hydrophobic bile salts prohibit bacterial proliferation (**a**). Bile and pancreatic juice flow prohibits bacterial invasion (**b**). The sphincter of Oddi, a muscle situated at the junction of the biliary tract and duodenum, controls the flow of bile and pancreatic juice; the high-pressure zone here prevents reflux of bacteria in the duodenum into the biliary tract (**c**). The gut-vascular barrier also controls the translocation of antigens and thus prevents the translocation of bacteria from the gut through the portal vein to the liver or pancreas (31)(**d**).

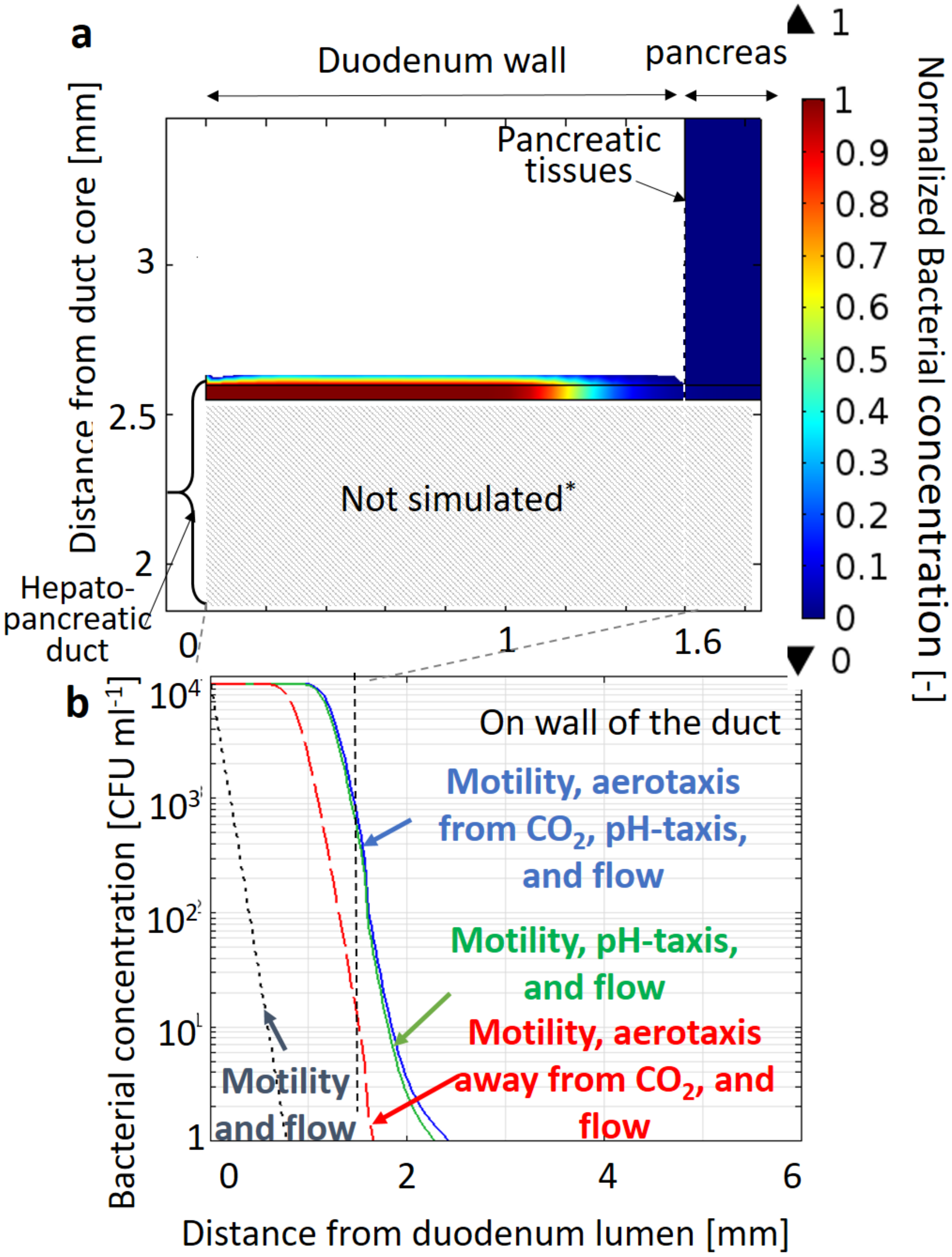

Figure S2. Migration of aerobic bacteria into healthy pancreas is driven by pH-taxis and aerotaxis away from carbon dioxide, under the pH- and CO_2-gradients at the junction of duodenum and pancreatic duct.*shaded area is not simulated in order to solve diffusion-convection equation with finest mesh selection.

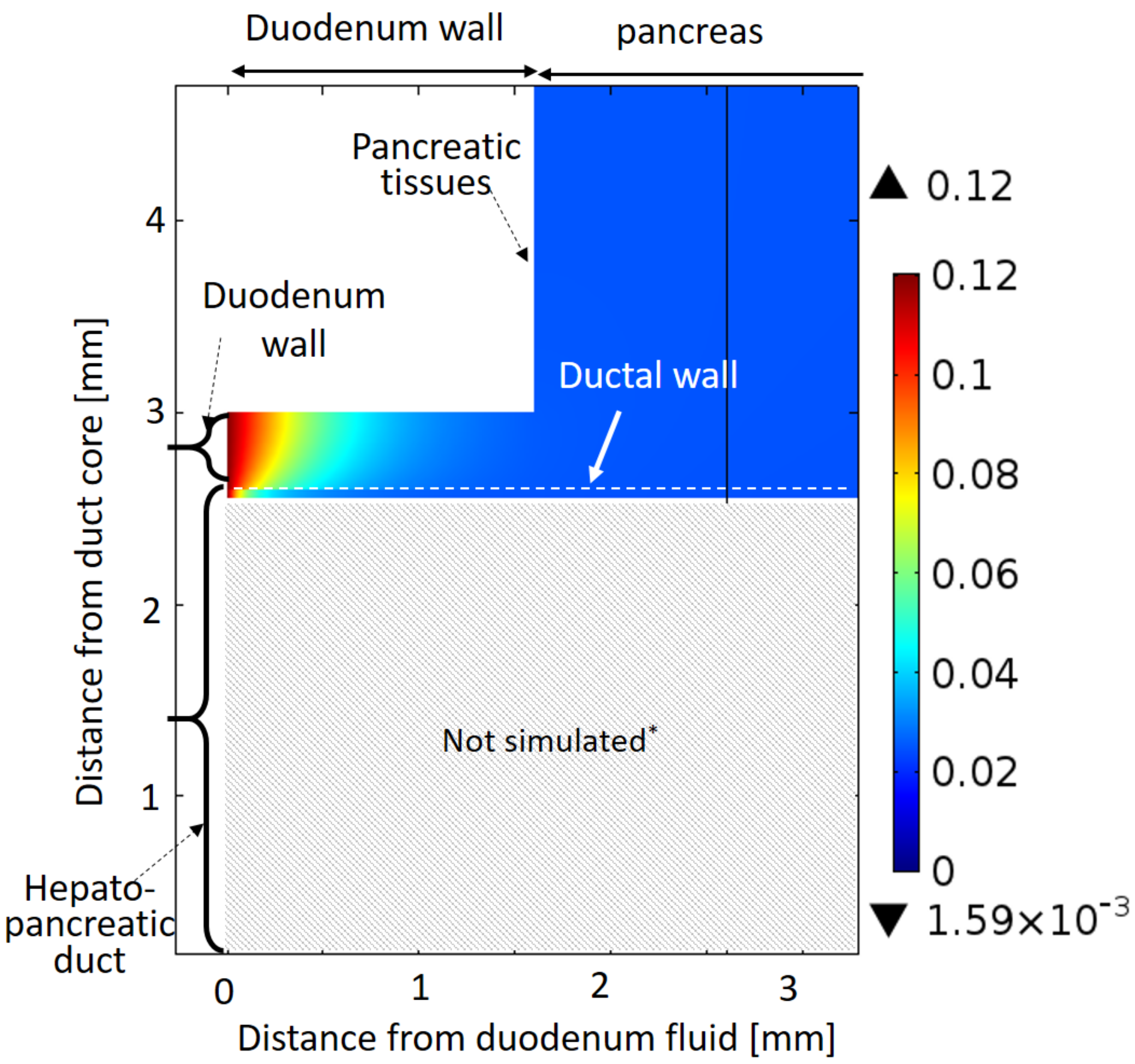

Figure S3. Simulated oxygen concentration in duodenum fluid is higher due to oxygen availability from air but low in pancreas with tumor due to hypoxia.

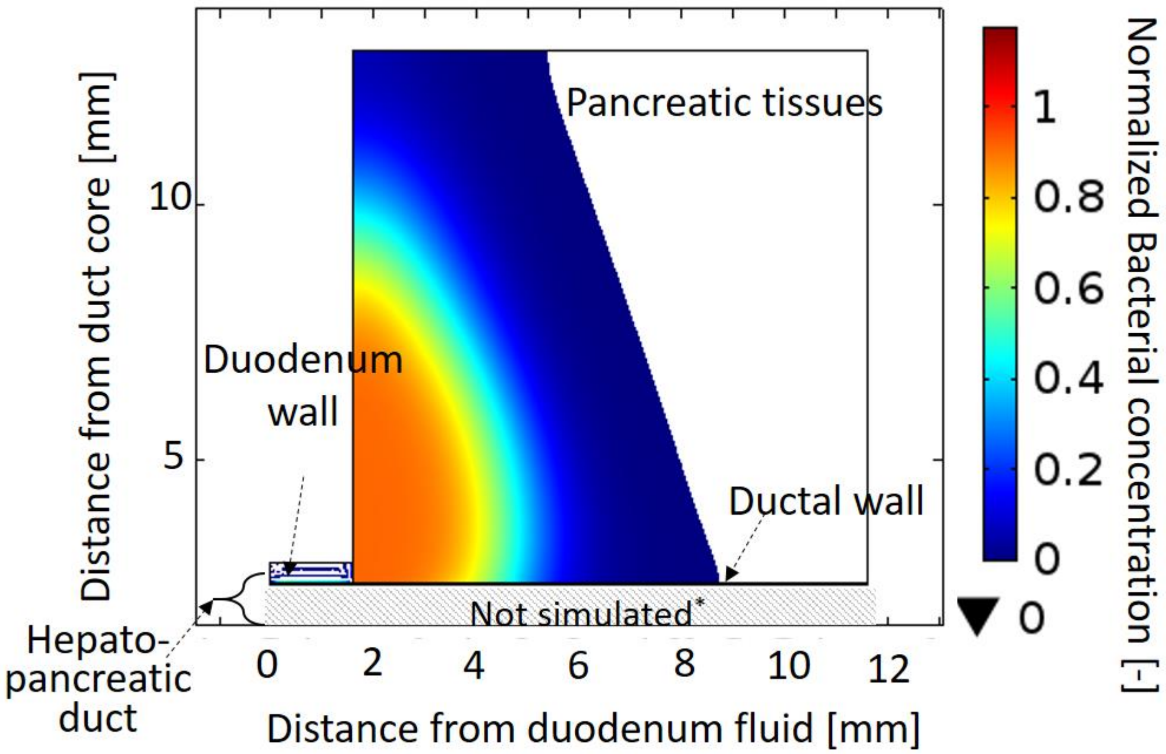

Figure S4. Simulated bacterial concentration in pancreas with tumor. Extended version of figure 4a.

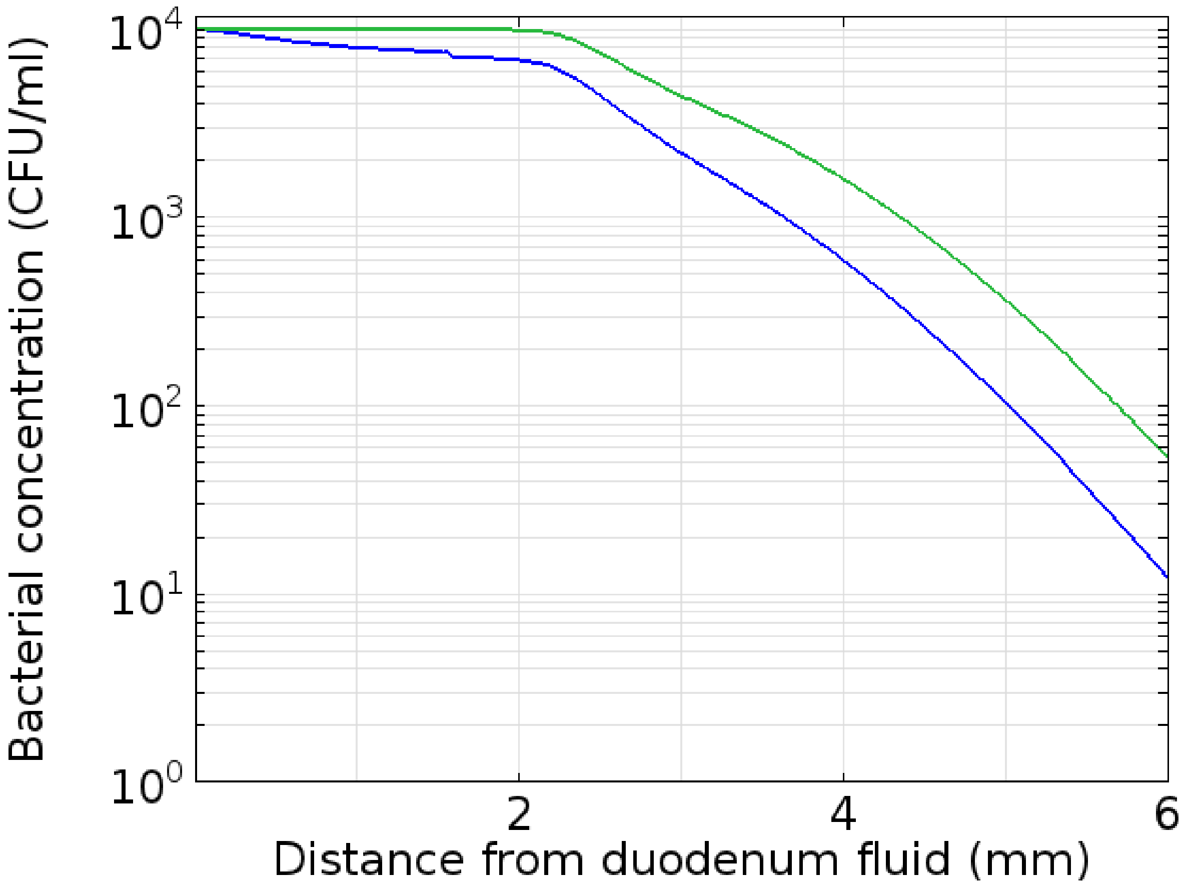

Figure S5. Bacterial migration into pancreas with tumor with aerotaxis to higher oxygen (blue) and without that (green). Aerotaxis of aerobic bacteria attracts them to higher oxygen at duodenum.

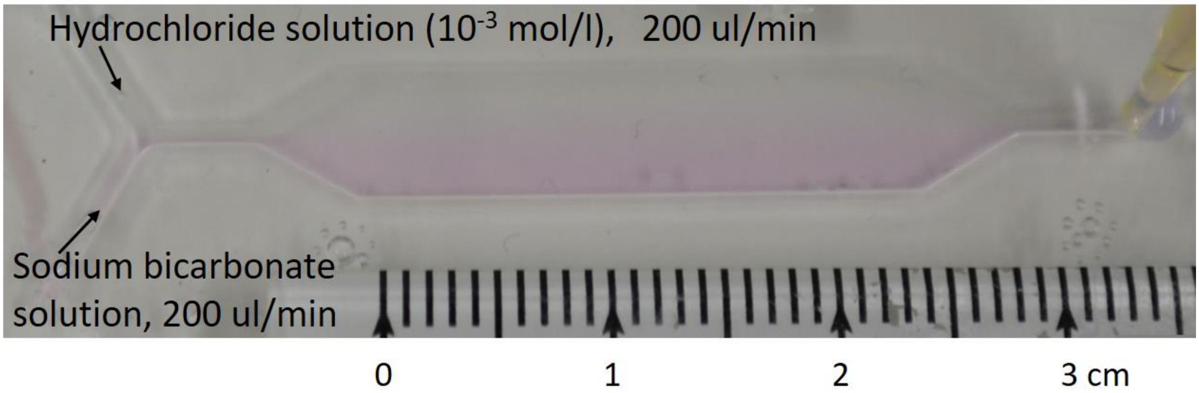

Figure S6. The pH in a microfluidic channel was visualized in phenolphthalein solution. The pH increased from top at 4–5, at middle in colorless at pH 5–7, to the bottom at alkaline in pink. Hydrochloride solution at pH 3 and sodium bicarbonate at 80 mmol/l were poured from top and bottom inlets, respectively.

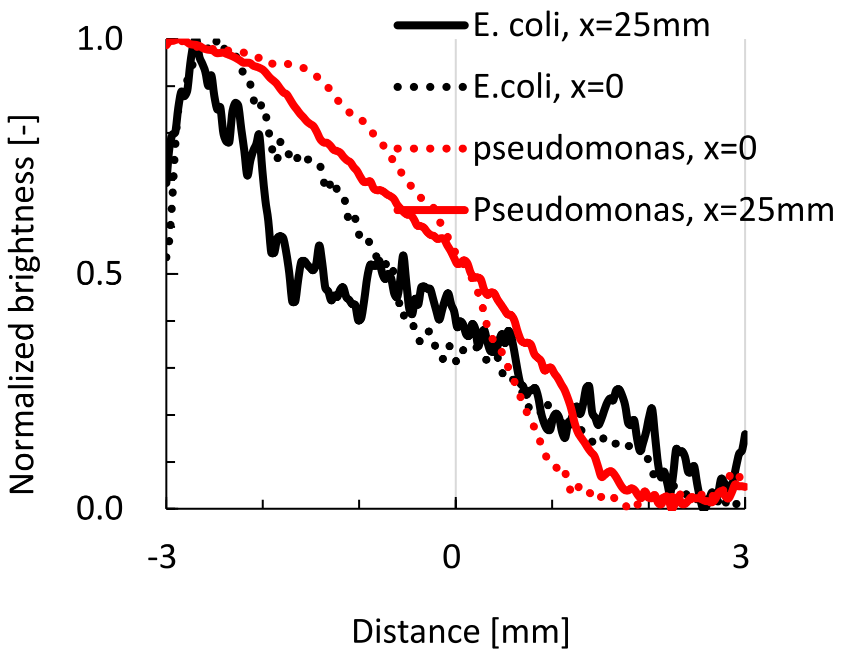

Figure S7. Comparison of bacterial distribution in the microfluidic channel with pH-gradient for *GFP E. coli* (black) and *Pseudomonas fluorescens* (red). Bacteria were poured with bicarbonate solution at the bottom inlet, while the other inlet was hydrochloride solution at pH 2.9.

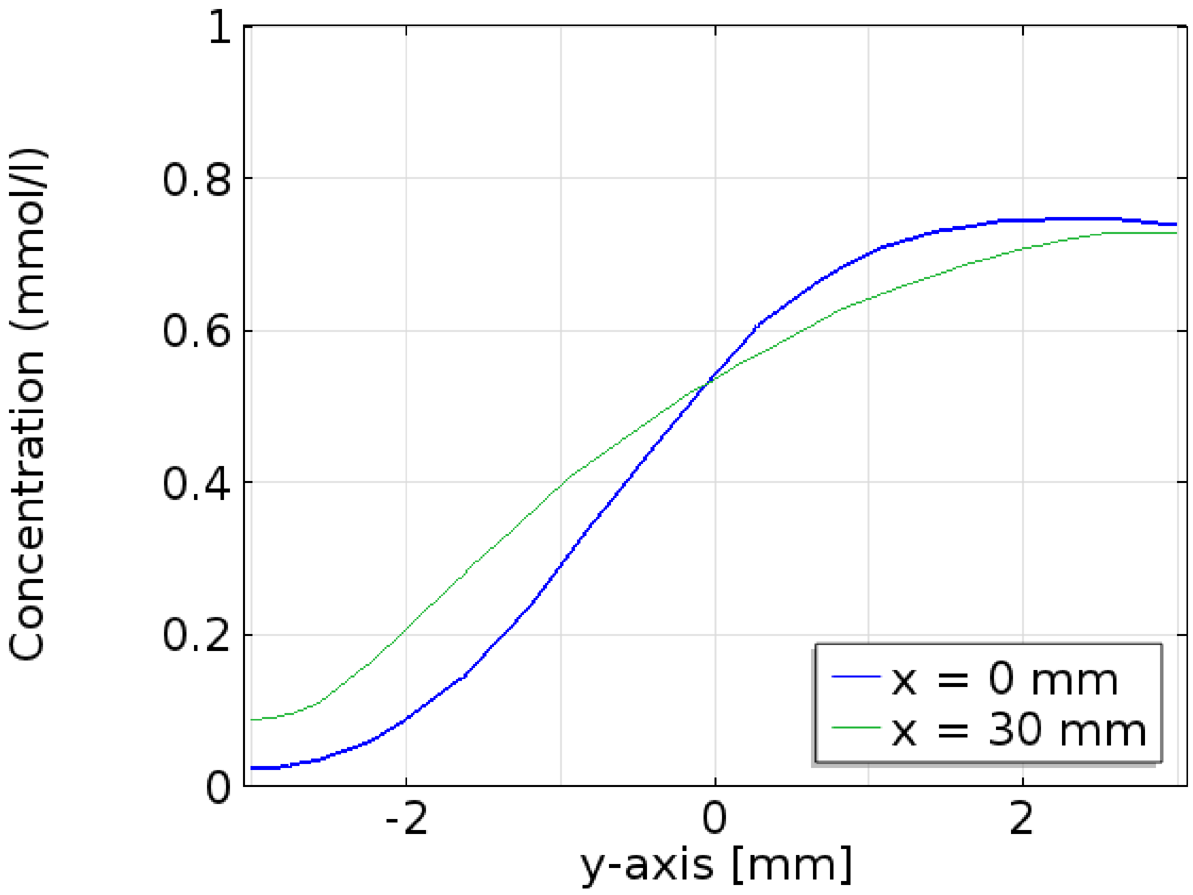

Figure S8. Simulated carbon dioxide concentration in a microfluidic channel is higher in the upper channel, due to a neutralization of hydrogen ion by bicarbonate.

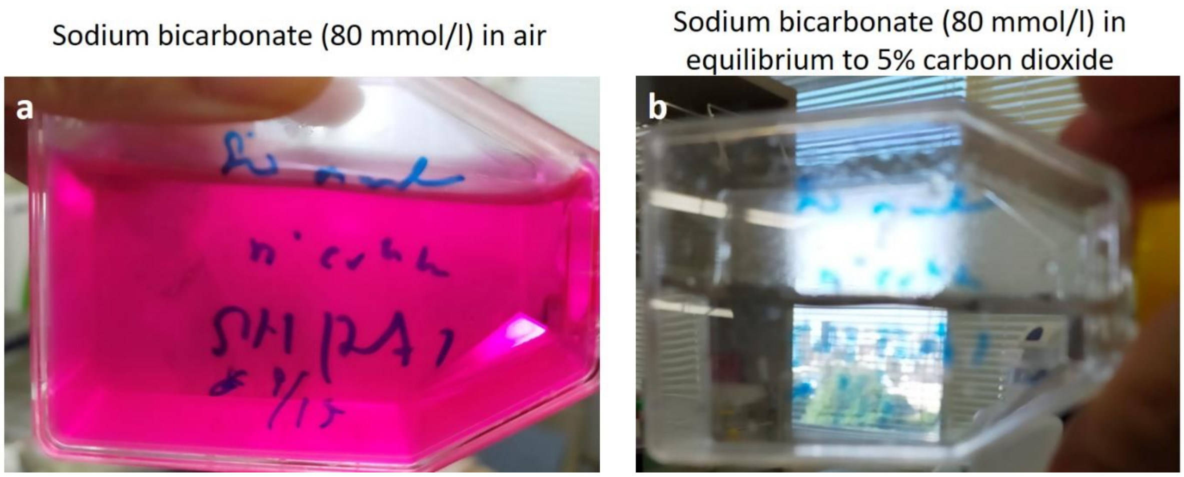

Figure S9. Sodium bicarbonate in in equilibrium to air (a) and 5% carbon dioxide (b), visualized in phenolphthalein solution. Under the carbon dioxide level at pancreas, pH of bicarbonate at 80 mmol/l drops at neutral or slightly alkaline.

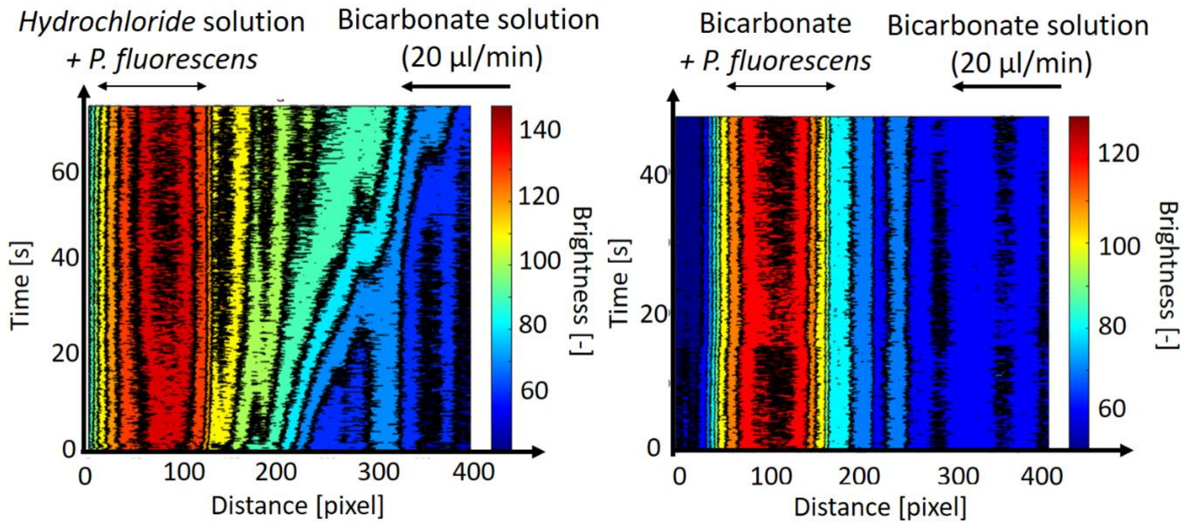

Figure S10. Measured migration of *P. fluorescens* against flow of bicarbonate, in equilibrium to 5% carbon dioxide, at 52 µm/s maximum fluid velocity, from hydrochloride at pH 5–6 (a) and bicarbonate solution (b). *P. fluorescens* in acid solution penetrated against flow approximately 60 µm/s due to pH-taxis (**a**), though *P. fluorescens* did not migrate against flow from the bicarbonate solution due to motility alone (**b**).

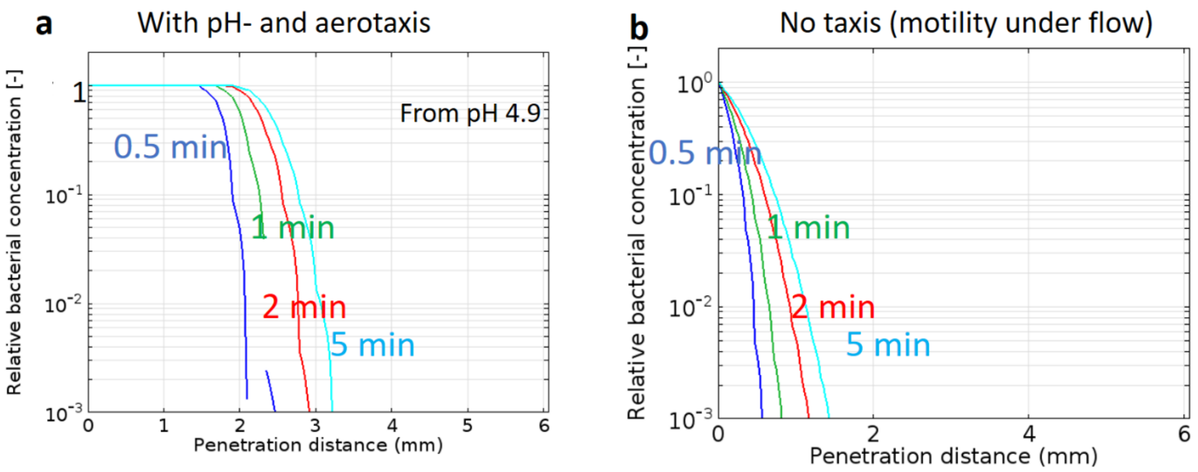

Figure S11. Simulated migration of aerobic bacteria from duodenum to pancreas with flow rate of 20 μl/min, the same as experiment in T-shaped cylinder. **a**: migration from acid pH under bicarbonate flow, **b**: migration from neutral pH.

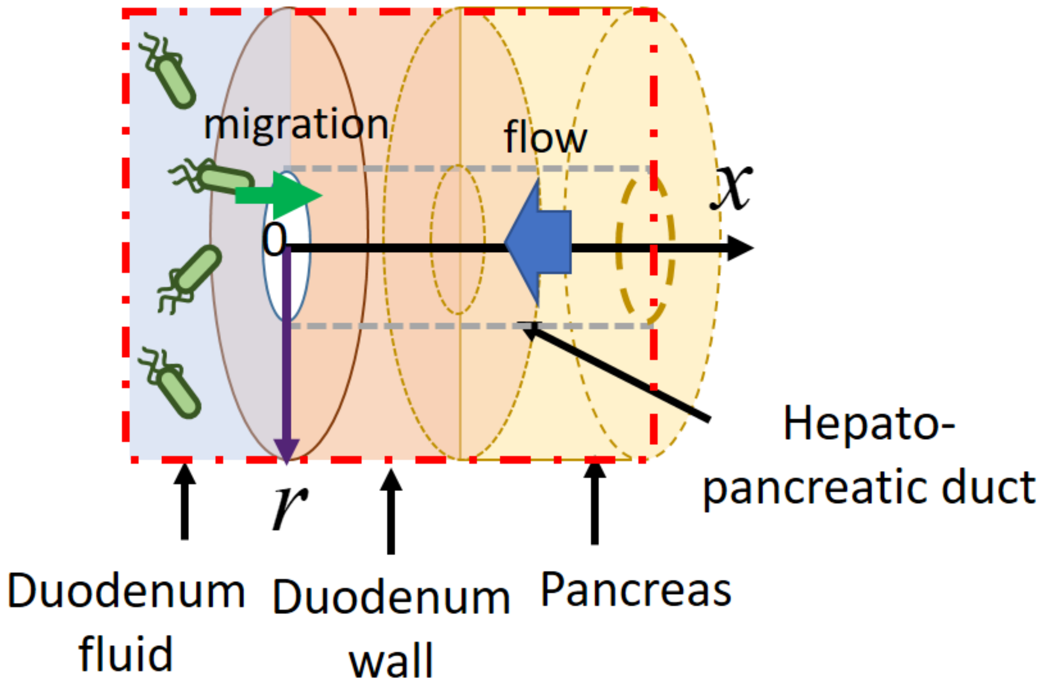

Figure S12. A geometry used in the modeling. Hepato-pancreatic duct is modeled as symmetric cylinder.

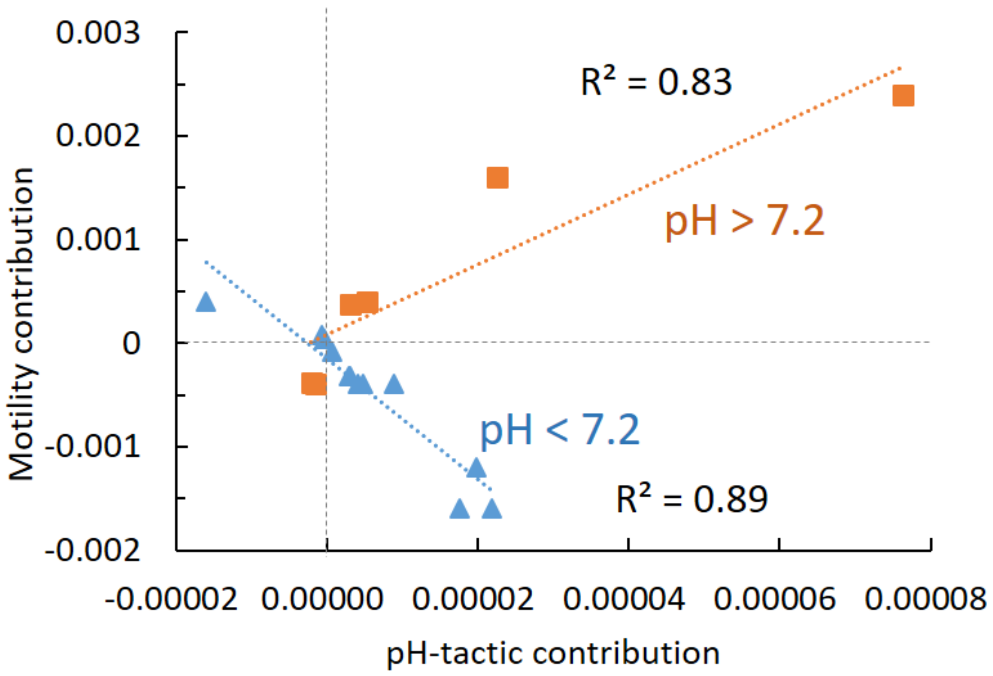

Figure S13. Motility contribution of *Serratia marcescens* in steady state in the data of Zhuang and wo-workers (2015) correlated reasonably well to pH-tactic contribution. pH-tactic contribution is calculated in Eqn. (9). Blue triangles indicate data of pH < 7.2 and orange squares indicate pH > 7.2.

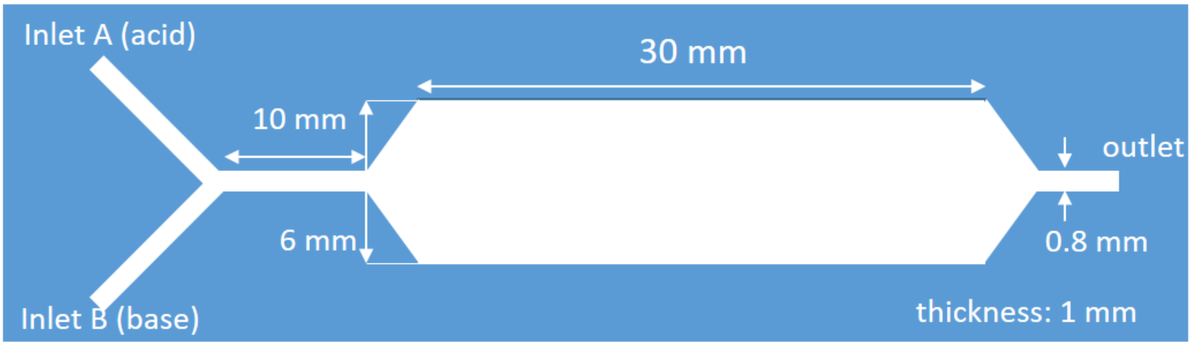

Figure S14. Configuration of a microfluidic device that can generate a steady pH gradient, used for analysis of bacterial pH-taxis.

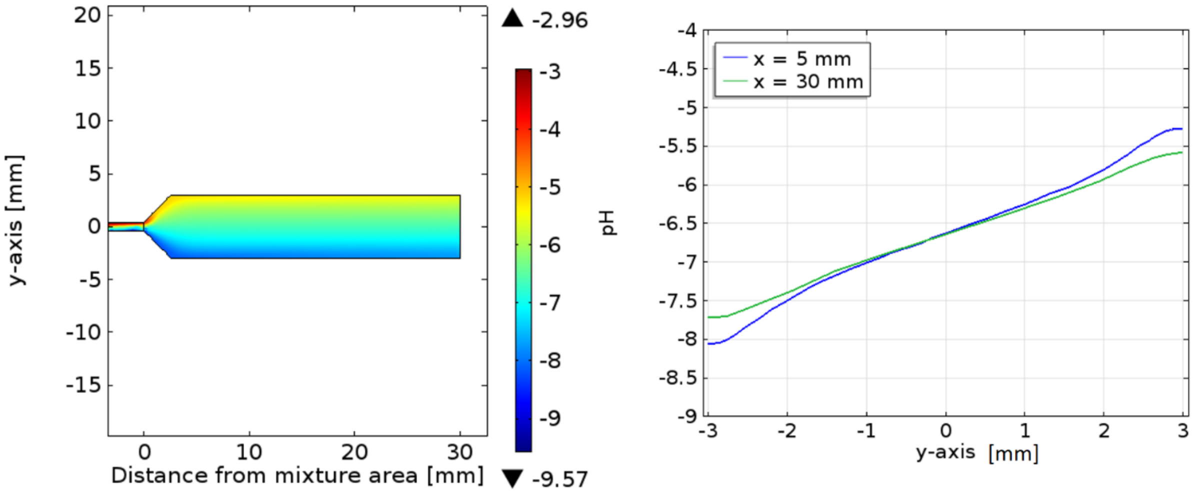

Figure S15. Simulated pH distribution in a microfluidic channel.

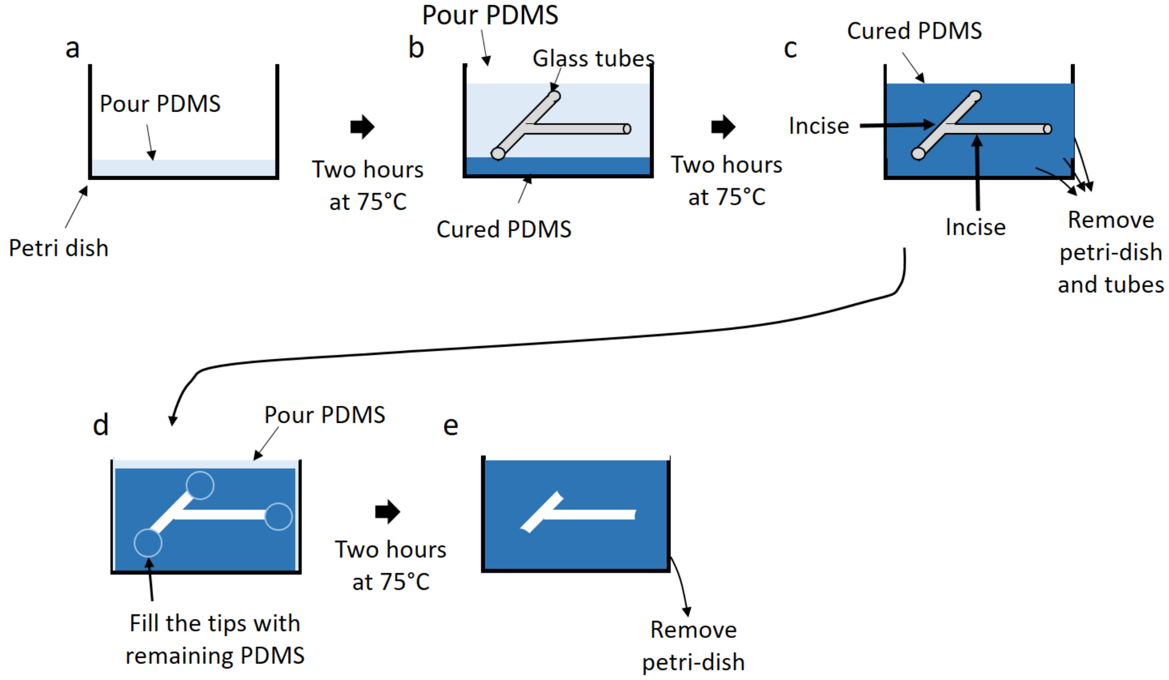

Figure S16. Fabrication process of a T-shaped microfluidic device.

.

Supplemental materials and methods

Validation of pH-tactic model

The pH-tactic model was validated against the measured distribution of *Serratia marcescens* under a pH gradient by Zuang and coworkers with their permissions(63). Motility and pH-tactic contributions were calculated from the difference in distribution density of bacteria for motility contribution and eqn. (9) for pH-taxis, respectively. The optimal pH for *S. marcescens*, pH_0 at 7.2, is used from the study. In the steady state, the ratio of motility to pH-taxis contribution is constant, as described in the following equation:

$$\underbrace{\frac{db}{dx}}_{\text{motility contribution}} = \underbrace{-\frac{\chi_0^{pH}}{\mu} \cdot \frac{1}{\left([H^+]-[H^+]_{opt}\right)} \cdot \frac{d\left([H^+]\right)}{dx} b}_{\text{pH-tactic contribution}} \qquad \text{(S1)}$$

This equation is obtained from eqn. (1) for the one-dimensional case without flow (*v* = 0) or aeortaxis ($\chi_0^{aero} = 0$). The motility and pH-tactic contributions are calculated from data in the literature (Figure S12) when the probability density of bacteria, ρ , was used in place of *b* in eqn. (S1). The contribution of motility was reasonably well correlated with the pH-taxis contribution in the literature (63) with correlation coefficients of 0.89 and 0.91 for pH < 7.2 and pH > 7.2 (figure S12), validating the model. The parameter of the chemotactic sensitivity coefficient, χ_0^{pH} [m^2 s^{-1}], was calculated from the slope of Figure S12, *k*, which is equal to the ratio of the chemotactic sensitivity coefficient to the random motility coefficient from Eqn. (10):

$$k = \frac{\chi_0^{pH}}{\mu_0} \qquad \text{(S2)}$$

These coefficients, *k*, are 65.1 and –35.2 for pH < 7.2 and pH > 7.2, respectively. From a given random motility coefficient, the chemotactic sensitivity coefficient of pH taxis is determined.

This model need to be carefully used in the regions where the pH is close to the optimal one ($h \approx h_0$) as the term, $1/(h-h_0)$, diverges. In this model, the simulated maximum pH in pancreas is at 7.6, which is lower than optimal pH at 8.

Mathematical modeling of transport of ions in hepato-pancreatic duct

Transport of ions in the duct that includes diffusion, flow, and reactions is mathematically modeled as follows:

$$\frac{\partial[H^+]}{\partial t} = \underbrace{D_{eff}^{H+}\left(\frac{\partial^2[H^+]}{\partial x^2} + \frac{\partial^2[H^+]}{\partial r^2} + \frac{1}{r}\frac{\partial[H^+]}{\partial r}\right)}_{\text{diffusion}} \underbrace{-\frac{\partial}{\partial x}\left(u_h[H^+]\right)}_{\text{bile and pancreatic juice flow}} \underbrace{-k_+[H^+][HCO_3^-] + k_-[CO_2]}_{\text{reactions}} \tag{S3}$$

Diffusion across the ductal wall at $r = r_h$ is described using permeability as follows:

$$Flux_{H^+} = P_{H^+}\left([H^+]_{r=r_h}^{wall} - [H^+]_{r=r_h}^{duct}\right) \tag{S4}$$

where P_{H^+} [m s^{-1}] is the ductal permeability of hydrogen ions. $[H^+]_{r=r_h}^{wall}$ and $[H^+]_{r=r_h}^{duct}$ [mol l^{-1}] are the hydrogen ion concentrations on the ductal wall in the duodenum and hepatopancreatic duct, respectively. The concentrations of bicarbonate and carbon dioxide in the duct, $[HCO_3^-]$ and $[CO_2]$ [mol l^{-1}], are also described in the same manner as:

$$\frac{\partial[HCO_3^-]}{\partial t} = \underbrace{D_{eff}^{HCO3-}\left(\frac{\partial^2[HCO_3^-]}{\partial x^2} + \frac{\partial^2[HCO_3^-]}{\partial r^2} + \frac{1}{r}\frac{\partial[HCO_3^-]}{\partial r}\right)}_{\text{diffusion}} \underbrace{-\frac{\partial}{\partial x}\left(u_h[HCO_3^-]\right)}_{\text{bile and pancreatic juice flow}} \underbrace{-k_+[H^+][HCO_3^-] + k_-[CO_2]}_{\text{reactions}} \tag{S5}$$

$$\frac{\partial[CO_2]}{\partial t} = \underbrace{D_{eff}^{CO2}\left(\frac{\partial^2[CO_2]}{\partial x^2} + \frac{\partial^2[CO_2]}{\partial r^2} + \frac{1}{r}\frac{\partial[CO_2]}{\partial r}\right)}_{\text{diffusion}} \underbrace{-\frac{\partial}{\partial x}\left(u_h[CO_2]\right)}_{\text{bile and pancreatic juice flow}} \underbrace{+k_+[H^+][HCO_3^-] - k_-[CO_2]}_{\text{reactions}} \tag{S6}$$

Oxygen transport

Oxygen transport in the hepatopancreatic duct, duodenal wall, and pancreatic tissues is mathematically modeled and includes diffusion and flow in the hepatopancreatic duct, as described in the following equation:

$$\frac{\partial a}{\partial t} = \underbrace{D_{eff}^{O_2}\left(\frac{\partial^2 a}{\partial x^2} + \frac{\partial^2 a}{\partial r^2} + \frac{1}{r}\frac{\partial a}{\partial r}\right)}_{\text{diffusion}} \underbrace{-\frac{\partial}{\partial x}\left(v_h a\right)}_{\substack{\text{bile and}\\ \text{pancreatic juice flow}}} \tag{S7}$$

where a [mol l^{-1}] is the oxygen concentration, D_{eff}^{O2} [m^2 s^{-1}] is the effective diffusion coefficient of oxygen in the hepatopancreatic duct, and pancreatic tissues are considered porous media. The effective diffusion coefficient of oxygen is thus described as:

$$D_{eff}^{O_2} = D_0^{O_2}\frac{\eta_w}{\eta_h}\cdot\frac{\phi}{\tau}\ (0 < r < r_h) \qquad \text{(S)}$$

D_0 [m^2 s^{-1}] is the diffusion coefficient of oxygen in water at 37°C, η_w [mPa·s] is the viscosity of water, η_h [mPa·s] is the viscosity of fluid in the hepatopancreatic duct, ϕ [-] is porosity and τ [-] is tortuosity.

Therefore, the flux of oxygen across the hepatopancreatic duct is also described as follows:

$$Flux = P_{O2}\left(a_{r=r_h}^{wall} - a_{r=r_h}^{duct}\right) \tag{S9}$$

Oxygen transport is not included in the healthy pancreas, assuming no oxygen concentration difference between healthy pancreas and duodenum, but was included for transport in the pancreas with tumor since oxygen concentration in the pancreatic tumor is lower due to rapid oxygen consumption by cancer cells

Simplified mathematical models of bacterial transport in one-dimension

$$\frac{\partial b}{\partial t} = \underbrace{\mu_{eff}\frac{\partial^2 b}{\partial x^2}}_{\text{motility}} - \underbrace{\frac{\partial}{\partial x}\left\{ \overset{\text{chemotactic sensitivity coefficient}}{\chi_0^a} \; \overset{\text{oxygen concentration}}{\frac{a}{\left(K_d^a + a\right)^2}} \cdot \overset{\text{concentration gradient}}{\frac{\partial a}{\partial x}} \; b \right\}}_{\text{aerotaxis to oxygen}} - \underbrace{\frac{\partial}{\partial x}\left\{ \overset{\text{negative aerotaxis}}{-} \; \chi_0^c \frac{1}{K_d^c + \underbrace{c}_{\text{carbon dioxide concentration}}} \cdot \frac{\partial c}{\partial x} b \right\}}_{\text{aerotaxis away from carbon dioxide}}$$

$$- \underbrace{\frac{\partial}{\partial x}\left\{ \frac{\chi_0^h}{\left(h - h_0\right)} \cdot \frac{dh}{dx} b \right\}}_{\text{pH-taxis from acid toward neutral pH}} - \underbrace{\frac{\partial}{\partial x}\left(\overbrace{v_h \quad b}^{\text{flow velocity}} \right)}_{\text{flow}}$$

1. Motility

$$v_{motility} = -\mu_{eff}\frac{\partial b}{\partial x}$$

μ_{eff} [m^2 s^{-1}]: Random motility coefficient

b [CFU/ml]: bacterial concentration in hepato-pancreatic duct

2. Aerotaxis (Keller-Segel model)

χ_0^a [m^2 s^{-1}] is chemotactic sensitivity coefficient of bacterial aerotaxis *; a* [mol/l]: oxygen concentration; K_d^a [mol/l]: dissociation constant;

3. pH-taxis

h [mol/l]: hydrogen ion concentration; proportional to hydrogen ion concentration gradient;

This equation only includes pH-taxis from acid but not from alkaline as pH in duodenum is lower than neutral (i.e. pancreatic juice).

$$V_{pH}^{x} = \chi_0^{pH} \frac{1}{\left(h - h_0\right)} \cdot \frac{dh}{dx} = \chi_0^{pH} \frac{1}{10^{-pH} - 10^{-pH_0}} \cdot \frac{d\left(10^{-pH}\right)}{dx}$$

$$pH = -\log_{10}(h)$$

Flow

v_h [m/s]: fluid velocity in hepato-pancreatic duct

When Reynolds number is lower (<2000), fluid flow is laminar flow, where fluid velocity in the cylinder follows

$$v_h\left(r\right) = v_{\max}\left\{1 - \left(\frac{r}{r_h}\right)^2\right\}$$

Maximum flow velocity in the cylinder, v_{max} [m s^{-1}], is in:

$$v_{\max} = \frac{2Q_h}{\pi r_h^{\,2}}$$

Q_h [m^3/s]: fluid flow rate

Penetration velocity is calculated in a simple manner:

$$v = v_{motility} + v_{pH-taxis} + v_{aero\_co2} + v_{aero+\_o2+} - v_h(r)$$

When motility and aerotactic velocity are neglitible compared with pH-taxis, this can be re-written as:

$$v = v_{pH-taxis}(h) - v_h(r)$$

The pH-tactic velocity in Keller-Segel model is:

$$V_{pH}^{x} = -\frac{\chi_0^{pH}}{h} \cdot \frac{dh}{dx} = -\chi \frac{d\ln(h)}{dx} = -\chi \frac{\Delta \ln(h)}{\Delta x} = -\chi \frac{\ln(h_d) - \ln(h_p)}{\Delta x}$$

Assuming the pH of pancreatic juice at 7.6,

$\ln(h_p) = \ln(10^{-7.6}) = -17.5$

For the duodenal pH at 5, $\ln(h_d) = -11.5$,

Thus, $\ln(h_p) - \ln(h_d) = -6$

For 4.5, $\ln(h_d) = -10.3$,

$\ln(h_p) - \ln(h_d) = 7.2$

Thus decrease in duodenal pH from 5 to 4.5, for example, leads to a 1.2 times higher pH-tactic velocity.

Bacterial penetration into pancreas occurs when $v > 0$,. i.e.,

$$v_{pH-taxis}(h) > v_h(r)$$

Supplemental references

1. Geller, L.T. et al. (2017). Potential role of intratumor bacteria in mediating tumor resistance to the chemotherapeutic drug gemcitabine. Science, 357: 1156–1160.
2. Riquelme, E., Y.et al. (2019). Tumor Microbiome Diversity and Composition Influence Pancreatic Cancer Outcomes. Cell. 178: 795-806.e12.
3. Harwood, C. S., K. Fosnaugh, and M. Dispensa. *Journal of Bacteriology* 171, no. 7 (1989): 4063–66.
4. Ezenobi, N. O., and G. C Okpokwasili. *International Journal of Current Research* 8, no. 08 (2016): 37124–30.
5. Sharma, Divakar, Anjali Garg, Manish Kumar, Faraz Rashid, and Asad U. Khan. *Frontiers in Microbiology* 10, no. December (2019): 1–9.
6. Mitrea, Laura, and Dan Cristian Vodnar. *Pathogens* 8, no. 4 (2019): 2–11.
7. Vijayakumar, Saranya, Sangeetha Rajenderan, Shakti Laishram, Shalini Anandan, Veeraraghavan Balaji, and Indranil Biswas. *Frontiers in Public Health* 4, no. May (2016): 1–9.
8. Abbott, D. M., and S. Z. Sudo. *Infection and Immunity* 17, no. 3 (1977): 655–60.
9. Zilm, Peter S., Neville J. Gully, and Anthony H. Rogers. *FEMS Microbiology Letters* 215, no. 2 (2002): 203–8.
10. Carabarin-Lima A, et al. (2016) First evidence of polar flagella in Klebsiella pneumoniae isolated from a patient with neonatal sepsis. *J Med Microbiol* 65(8):729–737.
11. He G, et al. (1999) Noninvasive measurement of anatomic structure and intraluminal oxygenation in the gastrointestinal tract of living mice with spatial and spectral EPR imaging. *Proc Natl Acad Sci U S A* 96(8):4586–4591.
12. Sender, R., S. Fuchs, and R. Milo. (2016). Revised Estimates for the Number of Human and Bacteria Cells in the Body. PLoS Biol. 14: 1–14.
13. Senturk S, et al. (2012) Diameters of the common bile duct in adults and postcholecystectomy patients: A study with 64-slice CT. *Eur J Radiol* 81(1):39–42.
14. Hadidi A (1983) Pancreatic duct diameter: Sonographic measurement in normal subjects. *J Clin Ultrasound* 11(1):17–22.
15. Nylund, K., T. Hausken, S. Ødegaard, G.E. Eide, and O.H. Gilja. (2012). Gastrointestinal wall thickness measured with transabdominal ultrasonography and its relationship to demographic factors in healthy subjects. *Ultraschall der Medizin*. 33.
16. Testoni PA, et al. (2006) Main pancreatic duct, common bile duct and sphincter of Oddi structure visualized by optical coherence tomography: An ex vivo study compared with histology. *Dig Liver Dis* 38(6):409–414.
17. Amdursky N, Lin Y, Aho N, Groenhof G (2019) Exploring fast proton transfer events associated with lateral proton diffusion on the surface of membranes. *Proc Natl Acad Sci U S A* 116(7):2443–2451.

18. Cornell Waste Management Institute (1996). http://compost.css.cornell.edu/oxygen/oxygen.diff.water.html Accessed Oct. 1st, 2021.
19. Gharibans AA (2012) Modeling Diffusion in the Small Intestines.
20. Schulz KG, Riebesell U, Rost B, Thoms S, Zeebe RE (2006) Determination of the rate constants for the carbon dioxide to bicarbonate inter-conversion in pH-buffered seawater systems. *Mar Chem* 100(1–2):53–65.
21. Menolascina F, et al. (2017) Logarithmic sensing in bacillus subtilis aerotaxis. *npj Syst Biol Appl* 3.
22. Esteller A (2008) Physiology of bile secretion. *World J Gastroenterol* 14(37):5641–5649.
23. Pandol SJ. The Exocrine Pancreas. San Rafael (CA): Morgan & Claypool Life Sciences; 2010. Water and Ion Secretion from the Pancreatic Ductal System
24. Ohya, T., Murakami, E., Kougame, A., Numata, Y., and Hiraoka, M. (2013). Proposal of Endoscopic Lithotripsy for Common Bile duct stones based on pathogenesis of stone formation. Cholithiasis. *Ann Surgery* 238 (1), 97-102 in Japanese.
25. Carr-Locke DL (1980) Serum and pancreatic juice carcinoembryonic antigen in pancreatic and biliary disease. *Gut* 21(8):656–661.
26. Harada H, Ueda O, Kochi F, Kobayashi T, Komazawa M. Comparative studies on viscosity and concentration of protein and hexosamine in pure pancreatic juice. *Gastroenterol Jpn.* 1981 Dec;16(6):623-6.
27. Reinhart WH, Näf G, Werth B (2010) Viscosity of human bile sampled from the common bile duct. *Clin Hemorheol Microcirc* 44(3):177–182.
28. Huber, M.L. et al. (2009). New international formulation for the viscosity of H_2O. *J. Phys. Chem. Ref. Data*. 38: 101–125.
29. Zhuang J, Carlsen RW, Sitti M (2015) PH-taxis of biohybrid microsystems. *Sci Rep* 5.
30. Gerolami A, et al. (1989) Calcium Carbonate Saturation in Human Pancreatic juice: Possible Role of Ductal H+ Secretion. *Gastroenterology* 96(2):881–884.
31. Spadoni, I., E. et al. (2015). A gut-vascular barrier controls the systemic dissemination of bacteria. *Science*. 350: 830–834.
32. Guo, W., Y. Zhang, S. Guo, Z. Mei, H. Liao, H. Dong, K. Wu, H. Ye, Y. Zhang, Y. Zhu, J. Lang, L. Hu, G. Jin, and X. Kong. (2021). Tumor microbiome contributes to an aggressive phenotype in the basal-like subtype of pancreatic cancer. *Commun. Biol.* 4: 1–13.
33. Gaiser, R.A., A. Halimi, H. Alkharaan, L. Lu, H. Davanian, K. Healy, L.W. Hugerth, Z. Ateeb, R. Valente, C. Fernández Moro, M. Del Chiaro, and M. Sällberg Chen. (2019). Enrichment of oral microbiota in early cystic precursors to invasive pancreatic cancer. *Gut*. 68: 2186–2194.
