# Supplementary material for: pH-taxis drives aerobic bacteria in duodenum to migrate into the pancreas with tumors
Source: Sci Rep. 2022 Feb 2;12:1783. doi: 10.1038/s41598-022-05554-8 (PMC8810860; doi:10.1038/s41598-022-05554-8)
Supplement: Supplementary file 6 — Supplementary Information 2. [file 41598_2022_5554_MOESM6_ESM.pdf]

Supporting movies captions

pH-taxis drives aerobic bacteria in duodenum to migrate into the pancreas with tumors

Hiroaki Shirai[a*] Cocoro Ito[b], Kosuke Tsukada[a,b]

Movie S1 *P. fluorescens* in bicarbonate solution of 80 mmo/l against flow of bicarbonate at 20 μl/min.

Movie S2. Migration of *P. fluorescens* in hydrochloride solution of pH 5-6 against flow of bicarbonate at 20 μl/min.

Movie S3. Simulated migration of aerobic bacteria against flow without pH difference. Note vertical axis is in logarithmic scale.

Movie S4. Simulated migration of aerobic bacteria against flow of bicarbonate from acidic pH (pH at 4.9 at x=0).
